# Baroreceptor-Inspired Microneedle Skin Patch for Pressure-Controlled Drug Release

**DOI:** 10.34133/bmef.0044

**Published:** 2024-06-28

**Authors:** Jiahui He, Mengjia Zheng, Tianli Hu, Ya Huang, Jingyou Su, Chunyi Zhi, Xinge Yu, Chenjie Xu

**Affiliations:** ^1^ Department of Biomedical Engineering, City University of Hong Kong, Tat Chee Ave, Kowloon, Hong Kong SAR, China.; ^2^ Hong Kong Centre for Cerebro-Cardiovascular Health Engineering, Hong Kong Science Park, New Territories, Hong Kong SAR, China.; ^3^ Department of Materials Science and Engineering, City University of Hong Kong, Tat Chee Ave, Kowloon, Hong Kong SAR, China.

## Abstract

**Objective**: We have developed a baroreceptor-inspired microneedle skin patch for pressure-controlled drug release. **Impact Statement**: This design is inspired by the skin baroreceptors, which are mechanosensitive elements of the peripheral nervous system. We adopt the finger touching to trigger the electric stimulation, ensuring a fast-response and user-friendly administration with potentially minimal off-target effects. ** Introduction**: Chronic skin diseases bring about large, recurrent skin damage and often require convenient and timely transdermal treatment. Traditional methods lack spatiotemporal controllable dosage, leaving a risk of skin irritation or drug resistance issues. **Methods**: The patch consists of drug-containing microneedles and stretchable electrode array. The electrode array, integrated with the piezoconductive switch and flexible battery, provides a mild electric current only at the spot that is pressed. Drugs in microneedles will then flow along the current into the skin tissues. The stretchable feature also provides the mechanical robustness and electric stability of the device on large skin area. **Results**: This device delivers Cy3 dye in pig skin with spatiotemporally controlled dosage, showing ~8 times higher fluorescence intensity than the passive delivery. We also deliver insulin and observe the reduction of the blood glucose level in the mouse model upon pressing. Compared with passive delivery without pressing, the dosage of drugs released by the simulation is 2.83 times higher. **Conclusion**: This baroreceptor-inspired microneedle skin patch acts as a good example of the biomimicking microneedle device in the precise control of the drug release profile at the spatiotemporal resolution.

## Introduction

Microneedles (MNs) are emerging devices for transdermal/intradermal drug administration [1,2]. Their key advantages, including minimal invasiveness, precisely controlled delivery, and improved skin absorption, have promoted their application in delivering a wide range of therapeutics including small molecules [3,4], proteins, nucleic acids [5], and even cells [6]. MN devices could release drugs through either passive or active mechanisms. In passive delivery, MNs act as both skin disruptors and drug carriers, offering a predetermined release profile (instant or sustained) [7]. The active delivery involves the response of MNs to either endogenous or exogenous stimuli, providing the precise control of the drug release profile [8–10].

One notable exogenous trigger for MN devices is electric field (EF) [11,12]. When combined with electro-responsive materials, the EF-sensitive MN devices respond instantly to the electric signal, offering the sustained or pulsed drug release on demand [13–15]. For example, Kusama et al. [13] reported a solid polymer-based ion-conductive porous MN device powered by biobattery for transdermal drug delivery and skin interstitial fluid extraction. The porous structure was coated with a layer of charged hydrogel that promoted drug release from MNs via electroosmotic flow. Our team also reported an implantable and bioresorbable MN device that was activated wirelessly for electrostimulation and sustainable delivery of anti-inflammatory drugs for muscle regeneration [16]. While these EF-responsive MN devices show excellent control over the drug release profiles, we also realize that these devices only provide a universal release pattern across the patches. This is an advantage for MN patches that deliver drugs for systemic diseases or treat local diseases affecting large area. However, it is limited for treating site-specific diseases (e.g., the itching problem on or around the burn) [17,18].

To realize the site-specific delivery, the electronics covering a large skin area should mechanically match the skin strain. Conventional electronic integration consists of rigid elements, is uncomfort, and may be loose when there is skin deformation; thus, it is not suitable to large-scale skin integration [19]. Flexible electronics maybe a promising solution to this issue. At present, some MN-based sensing and therapies have induced flexible/stretchable electrode design to improve the deformability of the device on skin [20–22]. For example, Lee et al. [22] reported an MN device for thermoresponsive release of insulin. However, the thermal field was not spot-specific controllable as it was generated by an interconnected electrode network. Wang et al. [10] reported a light-triggered MN for synergistic treatment of melanoma. However, the integration of a large number of light-emitted/controlled elements increased the complexity and cost of the device.

**Fig. 1.**
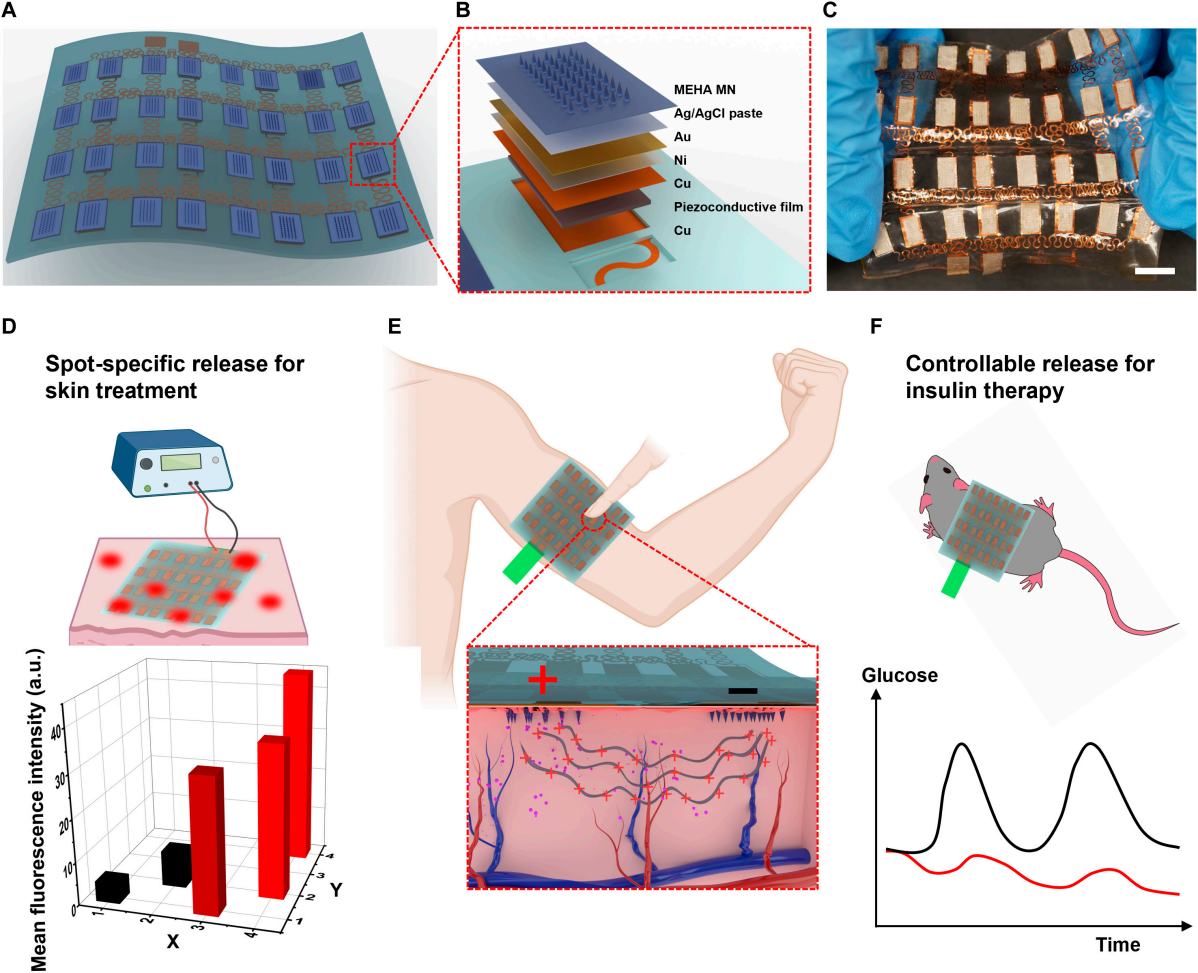
The baroreceptor-inspired MN skin patch. (A) Device. (B) Anode MN structure. (C) Device under stretching (scale bar: 1 cm). (D) Pressing-controlled release using the device (red bar is the fluorescence at touching spots, while black bar is the untouched sites). (E) Working mechanism. (F) Insulin delivery in the mouse model.

Here, we present an MN skin patch for spot-specific drug release upon pressing (Fig. 1). The design is inspired by the skin baroreceptor, which is the mechanosensitive element of the peripheral nervous system [23]. These receptors massively distribute on the skin epidermal layer and act as triggers for unconscious response toward dangerous stress/temperature. We adopt the finger touching to trigger the electric stimulation, ensuring a fast-response and user-friendly administration. Specifically, this baroreceptor-inspired MN skin patch (Fig. 1A) is composed of a double-layer structure, including MNs (Fig. 1B) and a stretchable electrode array (Fig. 1C). The electrode array consists of 2 layers (Fig. S1), and each layer is equipped with 16 cathodes and anodes. Each anode is integrated with the piezo-conductive film coupling with external copper film as an independent baroreceptor under the MNs. In contrast, the cathode has no piezo-conductive film and external copper sheet. To inhibit the electrochemical corrosion, both the copper films of the anode and the cathode are coated with composite anti-corrosion layers (Ni/Au and Ag/AgCl) (Fig. 1B). MNs are made of biocompatible and swellable polymers [i.e., methacrylated hyaluronic acid (MeHA)], which contain the model drugs (e.g., Cy3). The electrodes are attached to the backing layer of MNs using the compont adhesive (*n*-butyl α-cyanoacrylate mainly). The device can be powered by either a signal generator or a battery, providing a mild current of approximately 0.2 to 1 mA across the skin at the point of pressing (Fig. 1E). Based on the electroosmosis theory, the drugs flow with the current from MNs into the skin tissue. Compared with passive delivery without pressing, the dosage of drugs released by the EF simulation is 2.83 times higher (Fig. 1D). Finally, as a proof of concept, this device is used to deliver the insulin for managing the glucose level in the mouse model (Fig. 1F).

## Results

### Confirmation of the EF-controlled drug release from MNs in the hydrogel model for both micromolecules and macromolecules

Before fabricating the baroreceptor-inspired MN skin patch, we confirmed the EF-controlled drug release from the MeHA MN patch. This was done by paring the blank MN patch and the drug-containing MN patch connected to a cathode and an anode, respectively (Fig. S2). There are 2 model drugs: Cy3 (627 Da) and Cy3-insulin (7,700 Da). To ensure a constant pressure upon the MNs among all groups, the MN-attached anode and cathode were gently fixed on a gelatin hydrogel by a G-shaped clamp. The applied pressure of the clip was adjusted to a gentle level (0.5 N) by turning the bolt. The delivery efficiency or delivered drug dosage was derived by subtracting the remaining drugs in MeHA MNs from the original dosage.

For a duration of 20 min, the release rate of Cy3 under the current of 0.5 mA (active state) was ~2.8 folders of the control group (passive state) (Fig. S3A). This EF-controlled release is strongly influenced by the current intensity. As shown in Fig. S3B, during the same period (e.g., 15 min), the larger the current, the more the release of Cy3 from MNs. We also checked the release of a larger-size Cy3-insulin using the same setup. Under 2 mA, the release rate of Cy3-insulin was ~2.34 folders of the control group (Fig. S3C). Cy3-insulin’s release also depended on the current intensity (Fig. S3D). These experiments confirmed the capability of the MeHA MNs for EF-controlled release for both micromolecules and macromolecules.

### Fabrication and characterization of baroreceptor-inspired MN device

The electrode array is composed of 2 separated and layer-by-layer assembled patterns (cathode and anode, respectively) with 16 electrodes each (Fig. S1). The electrode patterns were fabricated using 10-μm-thick copper foil for its great conductivity. The copper foil was attached on the polydimethylsiloxane (PDMS) substrate, selectively etched as the first pattern via lithography, and sealed by another PDMS layer (100 μm thickness) (Fig. S4). Above the 100-μm PDMS layer, we fabricated the second electrode pattern using the same lithography and sealing process. The horizontal spacing and vertical spacing were 3 mm and 100 μm away, respectively, from the first pattern (Fig. S1). The serpentine design was taken to reduce the maximum strain on the rigid electrodes during the deformations. The cathode and anode electrodes were selectively exposed by laser cutting, leaving square cavities for MNs and zoflex piezoconductive film incorporation. We chose a low laser power (5 mA) here to minimize the damage of the electrode surface. Next, the zoflex films (4 mm × 8 mm × 50 μm) were fixed onto the anode by conductive paste and fully covered by attaching copper sheet using compont glue. The zoflex piezoconductive film was an elastic and porous rubber mixing with conductive components. It was electrically insulated (>30 MΩ) originally but became conductive (0.4 to 1.5 Ω) when a pressing toward the anode electrode was applied on the film.

MNs were fabricated through template molding using MeHA as the construction material and insulin as the model drug. Each MN patch (1 cm × 1 cm; Fig. S5A) had 100 MN tips with a height of ~700 μm, a base width of ~250 μm, an interneedle spacing of ~450 μm, and a tip diameter of 5 to 8 μm (Fig. S6). The methacrylation degree of MeHA was 80% [24]. The morphology of needle tips replicated well to the original MN template (Fig. S5C and D). The mechanical properties of the MeHA MN patch were examined by mechanical compression test (Fig. S5B). The MN tips could endure an average force of 0.1 N per needle, which is higher than the force of 0.058 N required for MN insertion into skin [5].

The next step is to combine the MN patch with the electrode array (Fig. S4). The MN patch was cut into 2 pieces (4 mm × 8 mm in size, 5 × 10 array), each of which was attached onto the cathode or anode, respectively. There might have some contact between the electrodes and the skin interstitial fluid (extracted by the MeHA MNs during the skin insertion), leading to electrochemical corrosion. To avoid this, we deposited a composite anti-corrosion layer (Ni/Au and Ag/AgCl) on each cathode and copper sheet on the anode, before the MN integration (Fig. 1B). The compont adhesive was dip-coated as the adhesive layer for electrode and MN attachment. The compont glue, commonly used for surgical suturing, is biocompatible and biodegradable [25]. Its main component, α-cyanoacrylate, underwent rapid anionic polymerization with water vapor as initiator, providing stable adhesion between electrode and MNs. The thickness of the adhesive layer was minimized to ~6.5 ± 1.1 nm to reduce the potential influence to the resistance of the device. The scanning electron microscopy (SEM) images and energy dispersive spectrometer (EDS) analysis (Figs. S7 and S8) displayed the layer-by-layer structure and compositions. The Au/Ni layer was not labeled because of the interference from sputter-coated Au and impurity elements (Ni) from SEM platform.

### Skin penetration and piezoconductivity test of the MN device

The assembled device consisting of MNs and electrodes was powered using the electrochemical station in the in vitro test. The electrochemical station provided a stable constant current in the range of 0.2 to 1 mA. The MN device was thumb pressed on the fresh mouse skin. The histology analysis of the cryosectioned skin tissue revealed that MNs disrupted the stratum corneum and created a 200- to 300-μm penetration into the dermis layer (left, Fig. S9A). The fluorescence image suggests the diffusion of Cy3-insulin from the penetration site into the surrounding tissue (right, Fig. S9A). We also examined the change of skin resistance due to skin disruption. Through the multimeter, we found that the skin resistances of both pig and mouse skin were greatly reduced to 38.14% and 3.52% of the original value, respectively, after the application of MN device (Fig. S9B). The great difference of reduction in the mice and porcine skin impedance should be due to the different features between porcine and mouse skin [26]. For instance, mouse skin is much thinner and loosely attached.

We further evaluated the pressure response of the device. The electrical stability of the device depends on the piezoconductivity and elasticity of the zoflex film. The MN device was mounted on the mouse skin by thumb pressing MN tips into the skin, and the adhesion force of PDMS ensured the subsequent attachment of the device on the skin. MN tips were allowed to fully swell in the skin for 5 min so the electrical impedance between the anode and cathode became stable. Then, an oscillator was applied to simulate the finger touching, providing a pulse force (~0.5 N) onto the anode electrode and subsequently a periodic current passing through the skin. Without the pressing from the oscillator, the measured current was negligible and the voltage stayed as the open-circuit voltage. The pressing from the oscillator raised the current (0.25 mA). The circuit became on and off along the oscillation (Fig. S9D to I). Besides the on–off cycle, we also examined the device by applying a constant pressure. Under a constant pressing and a constant voltage, the loading impedance between the anode and cathode was stable at ~12 kΩ, which was indicated by the great linearity between the loading current (<0.8 mA) and the loading voltage (<9 V) (Fig. S9C). We examined the morphology change of MNs before and after the penetration on pig and mouse skin. As shown in Fig. S10, all MN tips were intact after peeling off, except that the MNs did not stand as straight as before, which was due to the swelling of MNs in the skin.

### Anti-electrochemical corrosion property of the MN device

To minimize the electrochemical corrosion of the copper electrode during electric stimulation in skin tissue, we deposited a Ni/Au layer on both anode and cathode electrode surfaces. Ni/Au are well known for their excellent electrochemical stability and bonding strength on metallic materials [27,28]. We investigated the corrosion protection of the Ni/Au layer on the electrodes by comparing it with the uncoated and carbon-coated electrodes in a potentiodynamic analysis. Figure S11A and Table S1 show that the Ni/Au-coated electrode had higher corrosion potential (Ecorr) and corrosion current (Icorr) than both the uncoated electrode and the carbon-coated electrode. In the Nyquist plots (Fig. S11B), the carbon-coated electrode had similar curve with the uncoated electrode, indicating a similar ion conversion rate. The porous and loose carbon coating layer could not fully cover the copper surface. However, that of the Ni/Au-coated electrode had bigger radius, indicating a slower ion conversion rate, due to its dense surface and electrochemical stability. Additionally, we screen-printed a layer of Ag/AgCl paste onto the Ni/Au layer to further improve the electrochemical stability of electrode (Fig. S12).

### Stretchability of the MN device

**Fig. 2.**
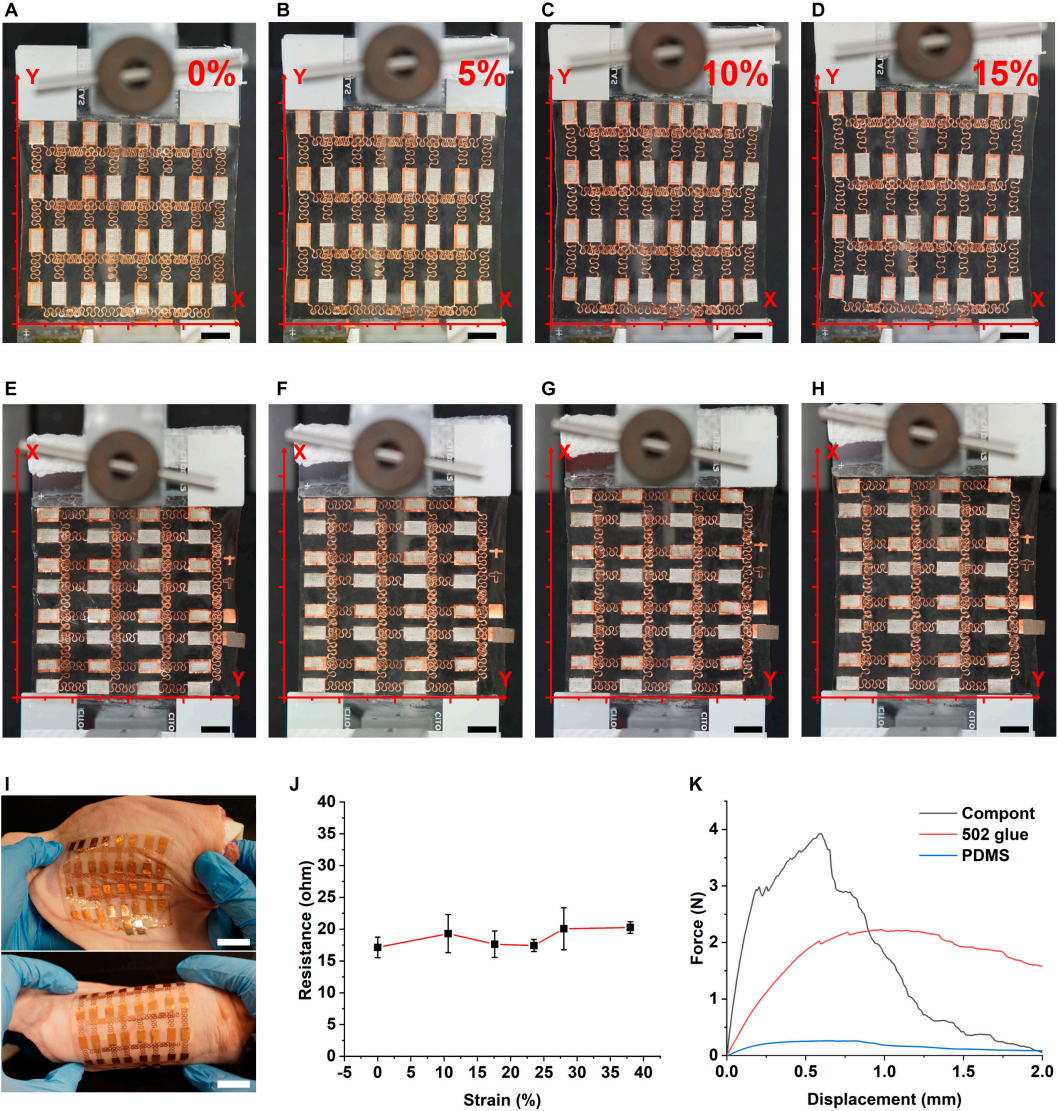
The stretchability and adhesion test of the electrode array. (A to D) Optical images of MN array under vertical stretching (scale bar: 1 cm). (E to H) Optical images of MN array horizontal stretching (scale bar: 1 cm). (I) Device under bending and twisting on the hairless pig skin (scale bar: 2 cm). (J) Electrical resistance between the anode side and the input side. (K) Adhesion force by 2 types of commercial glue: 502 (α-ethyl cyanoacrylate) or compont (*n*-butyl α-cyanoacrylate).

In view of the comfort of wear of the device and permitting adaption to different body curvatures of human beings [29], the electrode pattern of the MN array was designed with stretchable feature [30–32]. Dual-directional stretchability test was taken to examine this property (Fig. 2). The strain level is defined as the ratio of changing length to the original length of the whole device. As shown in Fig. 2 (A to H), the device was elongated to 115% of the original length and still maintained robustness in both directions. Furthermore, we applied the device on the hairless pig skin. Under bending or twisting, the device was fixed tightly on the skin surface (Fig. 2I).

We further evaluated the electrical stability of the electrode array under stretching. The electrical resistance between each electrode island only showed a slight increase of ~3.13 Ω under 38% strain (Fig. 2J), which is negligible compared to the resistance of human skin (40 to 150 kΩ) [13]. Therefore, this stretchable electrode layout has a relatively stable resistance under the strain.

Besides the stretchable layout, the interface between the PDMS substrate and the copper foil (the backpatch of the drug-loading MNs) is another key issue, as the adhesion should be maintained durable even under sweat environment or deformation. To optimize the adhesion between copper foil and PDMS, we prepared plasma-cleaned, flat PDMS substrate and copper foil, which were adhered together by 2 types of commercial glue: 502 (α-ethyl cyanoacrylate) or compont (*n*-butyl α-cyanoacrylate). As presented in Fig. 2K, in a 90° peel-off test, both compounds significantly improved the adhesion force, compared to van der Waals force provided by PDMS only. Especially, the compont adhesive had provided higher adhesion force before breakage. Finally, we selected compont glue to integrate MN and electrode due to its great flexibility and durable adhesion.

### Examination of the drug delivery capability ex vivo

**Fig. 3.**
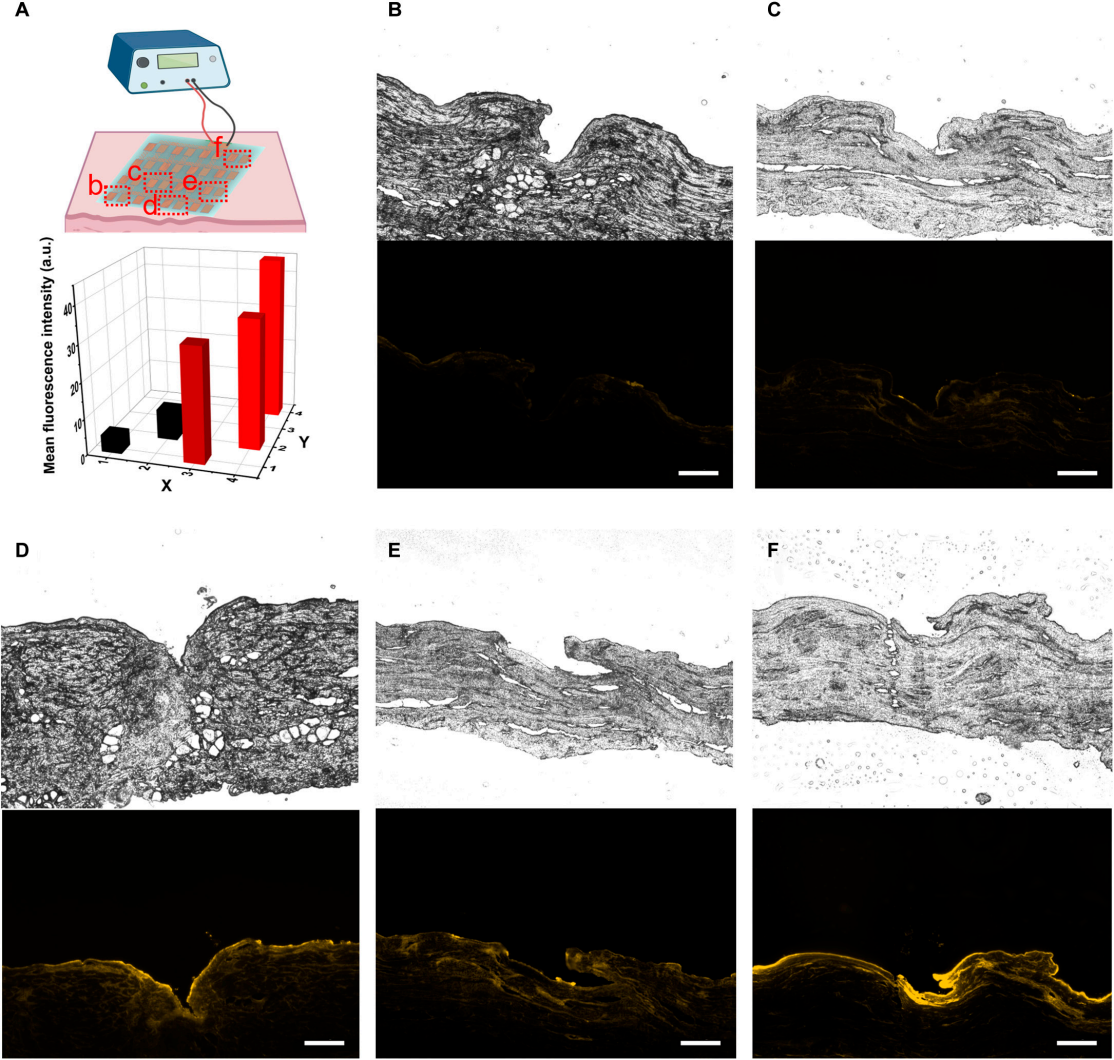
Pressing-induced Cy3-insulin release in pig ear skin. (A) illustration of the device operated via pressing (top) and the quantification of fluorescence in (B) to (F). Fluorescence images of pig skin after being applied with (B) 0 mA for 10 min, (C) 0 mA for 20 min, (D) 0.5 mA for 10 min, (E) 1 mA for 10 min, and (F) 1 mA for 20 min (scale bar: 200 μm).

The porcine ear skin was used to study the delivery capability of the MN device [26]. MN tips were first thumb-pressed into the skin before the device was connected with the electrochemical station (the power). To compare the difference of release performance between passive release and electric triggered release, 2 groups of experiments (with and without pressing to generate current) were carried out side by side. Without pressing, the fluorescence intensity in the skin was negligible, although the stratum corneum layer had been disrupted (Fig. 3B and C). For the pressing group, we applied a gentle thumb press (~0.5 N) to keep triggering the electric release. An adult thumb or index finger is large enough to cover each MN unit (8 mm × 12 mm). When there was the thumb pressing (10 and 20 min), the fluorescence in the skin layer was significantly improved (especially around the MN created micropores; Fig. 3D to F). The fluorescence intensity and distribution of Cy3-insulin in the skin dermis layer also increased with the current from 0.5 to 1 mA (Fig. 3B to E). Besides the impact of current intensity, the longer the pressing lasted, the more drugs were released in the skin (Fig. 3E and F). Similarly, the Cy3 penetration showed more significant improvement under EF, due to its lower molecular weight (Fig. S13).

We understand that the pressing itself might contribute to the increased release (Fig. S14). Therefore, with the same force holding the clip, we compared the drug release of the same MN device with and without the current. As shown in Fig. S14, although the pressing results in the increased release rate, the electric stimulation contributes to the most improvement of release rate.

### In vivo examination of the drug delivery capability of the MN device

**Fig. 4.**
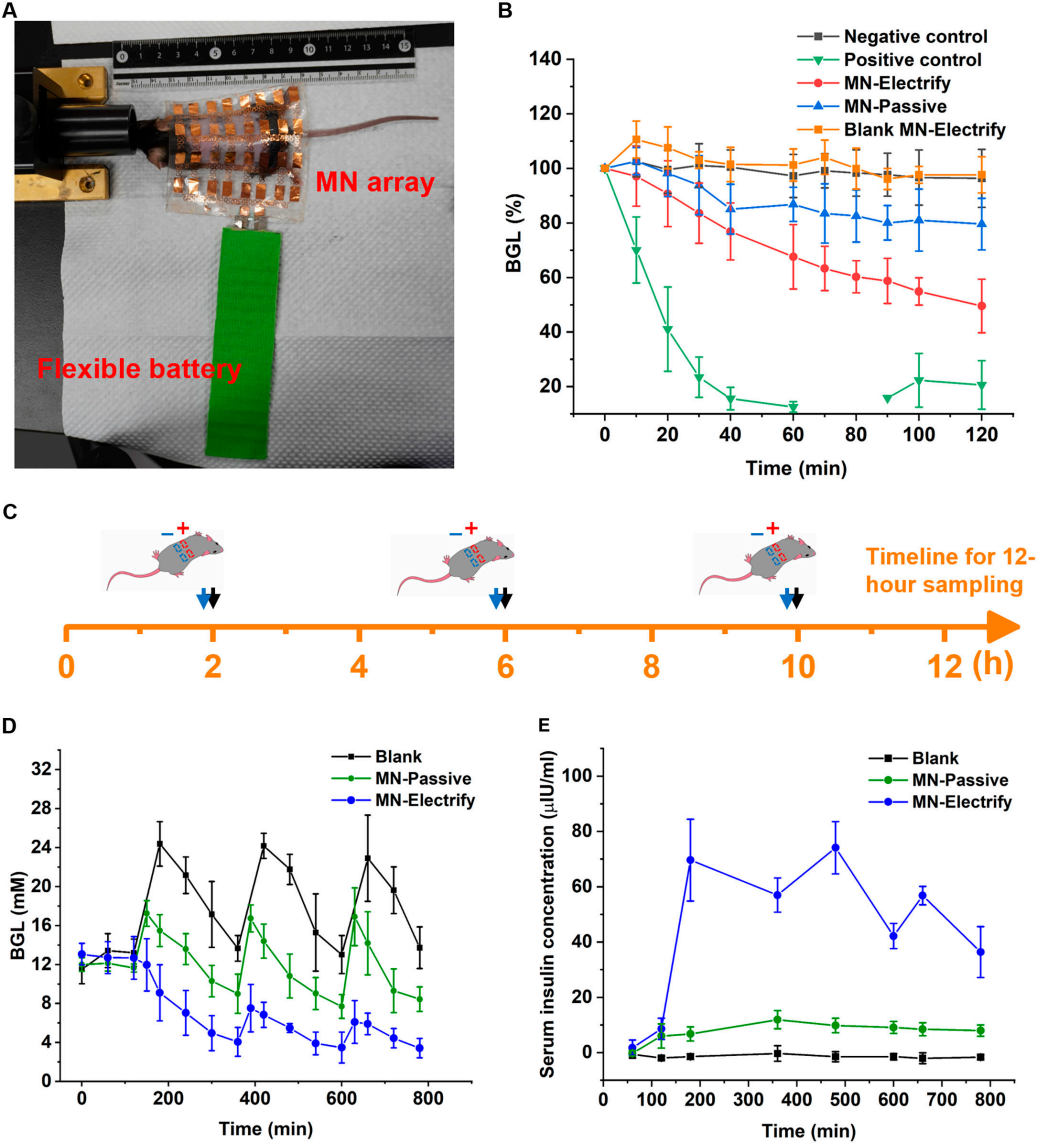
Pressure-controlled insulin delivery in the mouse model. (A) Mouse dorsum skin applied with the MN array powered by flexible battery. (B) BGL in the healthy mice during 2 h (*n* = 5). Negative control group: healthy mice applied with the device with blank MNs only; positive control group: intraperitoneal injection of 0.02 IU of insulin; MN-Passive group: constant passive release via 10 IU insulin-loaded anode MN; MN-Electrify group: electric-stimulated release via 10 IU insulin-loaded anode MNs. (C) Experimental plan for the periodic insulin delivery. Blue arrow: time point of electrify in the MN-Electrify group; black arrow: time point of glucose intragastric administration in all groups. (D) BGL in the periodic insulin delivery experiment during 13 h (insulin dosage: 20 IU; *n* = 5). (E) Blood insulin concentration in the periodic insulin delivery experiment during 13 h (*n* = 5).

To improve the portability and comfort of wear of the device, the battery is desired to be flexible, and recent advances of flexible battery give a great promise for the wearable medical device [33,34]. In this study, different from the above in vitro testing using the electrochemical station, the MN device was powered by a flexible battery in the animal experiments (Fig. 4A).

There were 5 experiment groups including negative control (blank group, assembled with one pair of blank MNs), positive control (intraperitoneal injection of 0.02 U of insulin), MN-Passive [one pair of MN electrodes (anode MN loaded with 10 IU of insulin and blank cathode MN) without being pressed], MN-Electrify (one pair of MN electrodes with 10-min electric stimulation via pressing), and blank MN-Electrify (one pair of blank MNs with 10-min electric stimulation via pressing). As shown in Fig. 4B, the blood glucose level (BGL) in the positive control group decreased rapidly within 20 min, while there was no change of BGL in the negative control group. For the MN-Passive group, BGL decreased slowly within 120 min. When there was electric stimulation via 10-min pressing (MN-Electrify group, pressure was stabilized by the same method in ex vivo skin model), the reduction of BGL (50% of the original glucose level) in 120 min was much faster than that of the MN-Passive group (80% of the original glucose level). Additionally, the blank MN-Electrify group showed no obvious decreasing of BGL compared to the negative control group, indicating that the electrify along had no effect on the BGL but only promoted the drug release from MNs.

We further carried out a periodic release of insulin on the mice for 13 h (Fig. 4C to E). To ensure the delivery efficiency, there were 2 pairs of MN electrode in each group; thus, the dosage of insulin was 20 IU. Through the whole experiment, BGL was measured every hour. At hour 1, the device was mounted on the mice. At hours 2, 6, and 10, the mice of all groups were gavaged with the glucose solution (Fig. 4C). There were 3 groups: MN-Passive, MN-Electrify, and blank group. For the blank group, there were 3 postprandial BGL peaks after the intragastric administration of glucose solution. When the MN patch was placed on the mice with thumb pressing (MN-Electrify, ~0.5 N, 0.5 to 0.7 mA), a significant reduction of glucose was observed. If there was only patch but without pressing (MN-Passive), the reduction of glucose would be minimal. In addition to BGL, we also checked the corresponding insulin level in the serum (Fig. 4E). In general, a higher insulin level was observed for the lower glucose stimulation. Taking the MN-Electrify group as an example, the insulin level maintained high level after the first EF stimulation. The microholes on mouse skin from the MN mounting recovered at 5 min after the removal of the device (Fig. S15).

## Discussion

MNs can provide long-term sustained release of drugs, which is usually achieved through the biodegradation of the MN matrix and/or the diffusion of drugs from the MN matrix to the skin tissue. While this sustained release is helpful for controlling chronic diseases, it is unsuitable for handling those situations where there are sudden changes of required drug dosage [7]. To address this need, this article reported a baroreceptor-inspired MN skin patch, which responded to the pressing for EF-triggered drug release. it was composed of drug-containing swellable MNs and stretchable electrode array. Swellable MNs were developed previously in our group, and the biocompatibility has been confirmed in cell and rabbit models [35,36]. Here, we also confirmed their suitability for the EF-controlled release for both micromolecules and macromolecules such as Cy3 and insulin (Fig. S3). Stretchable electrode array (for both anode and cathode) combined stretchable electrode pattern and flexible PDMS substrate, ensuring the wearability (Fig. 2) and stable electric signal in the experiment (Fig. 2J and Fig. S9). There were 16 pairs of MN-attached anode and MN-attached cathode, covering a size of 75 mm × 75 mm. A blank MN patch and a drug-containing MN patch connected to a cathode and an anode, respectively. This design was used for all the in vitro and in vivo experiment. However, in the mouse experiment, we only used (pressed) 2 gates due to the limited size of the mice. We carried out the drug release experiments in both ex vivo skin model (Fig. 3) and in vivo model (Fig. 4). Both confirmed the enhanced skin penetration of model drugs upon the finger pressing. The release and subsequent delivery of both Cy3 and Cy3-insulin from MNs under current are mainly based on electroosmosis, which produces bulk motion of the solvent itself that carries ions or neutral species with the solvent “stream.” However, the flow of electric current increases permeability of skin, which also contributes to the enhanced delivery [35,36].

As discussed in Introduction, this device is specifically designed for spot-specific drug release upon pressing. An ideal in vivo model would be some skin diseases like the itching problem on or around the burn, which eases upon the release of anesthetics. However, we do not have the access to this kind of animal models. Therefore, we chose the insulin as the model drugs and the healthy mice as the animal model. As shown above, the device clearly permitted the enhanced delivery of insulin upon the single (Fig. 4B) and periodic pressing (Fig. 4D). Through the quantification of delivered insulin in the blood (Fig. 4E), we realized that the release in the first pressing was the strongest (insulin concentration increased from 10 to 70 μU/ml). The second pressing only increased the insulin concentration from 60 to 75 μU/ml. The third press increased insulin from 45 to 58 μU/ml. This should be because of (a) the limited dosage of insulin and (b) the longer traveling distance for insulin moving from the MN matrix to the skin tissue. Specifically, as we showed in Fig. S3C, 10-min pressing would already release 60% the insulin from MeHA MNs under the current. Obviously, there was less insulin for the subsequent release (Fig. 4D). Insulin on or close to the MN surface should be more ready to move into the deep tissue under the current, in comparison to insulin buried in the MN matrix. In the periodic release experiment, the same 10-min pressing might not get the same amount of insulin out of MNs. During the animal experiment, we also found that the electric stimulation sometimes led to stress response of the mice. However, it should be negligible to human given that the current is <1 mA [37,38].

In the future, we plan to test this device in the minipig model for disease treatment of, i.e., psoriasis, vitiligo, or the itching problem on or around the burn, which requires safe, effective, and local delivery dosage. However, the complicated pattern of these lesions needs more dense, miniaturized MN units; thus, we will use advanced microelectromechanical system process to optimize the device. Besides, the long-term wearing brings about the concern of its breathability and adhesion. We think that ultrathin, porous substrate with great wet adhesion would ensure great comfort as well as stable performance even when sweating. Additionally, to realize point-of-care therapy, we would integrate microcontroller chip, software interface for accurate current regulation, and user-defined profile. Further, the device can also integrate with the commercial glucose monitoring system to achieve a reliable, closed-loop therapy.

## Materials and Methods

Sodium hyaluronic acid (HA; molecular weight, 300 kDa) was purchased from Freda Biochem Co. Ltd. (China). Methacrylic anhydride (MAA; 276685), *N*,*N*-dimethylformamide (DMF; 227056), phosphate-buffered saline (PBS) (1×), sodium chloride (S9888), and ferric chloride were purchased from Sigma-Aldrich. Sulfo-Cy3 NHS ester (627 Da) was purchased from J&K. Ag/AgCl ink was purchased from Julong Ltd., Shanghai. The compont medical adhesive was purchased from Compont Ltd., Beijing. Gelatin (from cold water fish) is purchased from Sigma. The electroplating solution and electroless plating solution were purchased from Pui Chen Ltd. SYLGARD 184 Silicone Elastomer Base (PDMS) was obtained from Dow Corning. Cy3-insulin power (7,700 Da) was purchased from Qiyue Biology Ltd., Xian. Compont adhesive smear type was purchased from Compont Ltd., Beijing. Human recombinant insulin and enzyme-linked immunosorbent assay (ELISA) insulin kit (Invitrogen) was purchased from Thermo Fisher Scientific Ltd. AZ 4620 and AZ 400K developer were purchased from AZ Electronic Materials Ltd., USA. All other materials were purchased from Sigma-Aldrich except as otherwise specified.

### Fabrication of MN patch

The stainless-steel master mold (300 μm base diameter, 5 μm tip radius, and 1,000 μm height) was purchased from Micropoint Technologies Pte. Ltd. (Singapore). The negative mold was made of PDMS (10 mm thick, Dow Corning 184 SYLGARD) to inversely replicate the master mold. This was done by pouring PDMS over the MN master structure, degassing by vacuum oven for 10 min, and curing the polymer at 70 °C for 1 h. MeHA was synthesized following our previous report [24]. Later, 500 mg of MeHA powder, 5 mg of photoinitiator (Irgacure 2959, 0.5 mg/ml), and 0.25 mg of Cy3 (627 Da) were dissolved in 10 ml of deionized (DI) water. Four hundred microliters of this mixture was poured onto the PDMS-negative mold, followed by centrifugation (4,700 rpm, 7 min) to remove any gas bubbles. For the Cy3-insulin loading, 0.164 mg of Cy3-insulin was dissolved in NaOH solution (pH 9 to 10) first, followed by adding photoinitiator and MeHA powder. Finally, the MN patch was fully dried in fume hood at room temperature before being peeled off. The MN patch was ultraviolet (UV)-exposed for 3 min (wavelength = 360 nm, intensity = 17.0 mW/cm^2^, model 30, OAI). All the drug-loaded MNs were stored at 4 °C, while blank MNs were stored at room temperature in a dry box.

### Device fabrication

A piece of 10-μm-thick copper foil was flattened by a cylinder on a spin-coated PDMS layer (1,500 rpm, 30 s) on a glass slide. Then, AZ 5214 photoresist (AZ Electronic Materials) was spin-coated on the copper foil at 3,000 rpm for 30 s and soft baked at 115 °C for 5 min. With the designed mask, the sample was exposed under a mask aligner (URE-2000/35AL deep UV, IOE, CAS) for 45 s. After the development in AZ 300MIF developer and post-baking for 5 min at 115 °C, the patterned copper foil was wet-etched in FeCl_3_ solution. Thus, the first layer of the electrode was finished and became the anode pattern. Later, we removed the photoresist on the pattern using acetone and by drying, spin-coated PDMS (30:1, 1,000 rpm, 30 s) on the pattern, and fully cured it at 70 °C for 1 h (PDMS as the intermediated layer). The second copper layer was attached to the PDMS surface and patterned through the same method as that of the first electrode and became the cathode pattern. We encapsulated the second pattern’s surface via spin-coated PDMS (1,500 rpm, 30 s) and fully cured it. Then, laser cutting was utilized to cut along the outline of anodes and cathodes on the PDMS surface. Afterward, we removed the cut area, leaving the exposed areas as anodes and cathodes for the MN integration. The device was finally cleaned with acetone, ethanol, and DI water in sequence.

### Electrode modification

To prevent the electrochemical corrosion on metallic electrodes, we connected the cathode pattern and a nickel plate with an electrochemical station. The cathodes were selectively electroplated with a Ni seed layer under 3 V, 20 °C for 10 s, followed by electroless nickel deposition (90 °C for 1 h). Then, the gold layer was coated on the Ni layer by electroplating under 7 V, 70 °C for 20 s (the device was washed with DI water and fully dried after each step). Finally, Ag/AgCl paste was coated on the Au layer via screen printing and then cured at 100 °C for 10 min to get the composite anti-corrosion layer (Ni/Au and Ag/AgCl). Using the same method, we gained a surface-modified copper foil and then cut it into copper sheets (5 mm × 8 mm, slightly bigger than cavity) by laser cutting. The zoflex film was attached onto the anodes using the conductive paste, which was then cured under 70 °C for 1 h. The copper sheet was attached onto the anode using the compont adhesive.

### Device assembly

The drug-loaded MN patch and the blank MN patch were attached onto the anode and cathode, respectively, using diluted compont adhesive (10% in acetone, 2 μl). For animal experiment used, we integrated the device with a flexible battery using the conductive paste. Finally, the device was stored in the dry box at room temperature.

### Surface morphology and mechanical characterization

The surface morphologies of MeHA MNs were characterized by field emission SEM (FEI Quanta 450 FEG SEM). The mechanical strength of MNs and stretchability of electrode array were measured by Instron 5942 Micro Tester in compression and tensile mode, respectively (strain rate of 0.5 mm/min).

### In vitro release test

Gelatin hydrogel prepared using 3% gelatin in 1× PBS solution was used as the skin model, and Cy3 and Cy3-labeled insulin were used as model drugs. A pair of electrodes integrated with drug-loaded MN and blank MN were inserted inside the hydrogel. The anode and cathode were connected to the CHI760 electrochemical station (using chronopotentiometry method) to perform EF-controlled release test. MNs were allowed to swell for 3 min before the power of electrochemical station was on. After the release, the MeHA MNs were put into 600 μl of DI water and kept at 4 °C overnight. Then, the solution was under centrifugation for 5 min under 3,000 rpm and the supernatant was collected to quantify the remaining model drugs in MeHA MNs. The fluorescence intensity of supernatant and standard Cy3/Cy3-insulin solution was quantified with a microplate reader (MD SpectraMAX M5e) (Cy3: excitation wavelength was 550 nm, emission wavelength was 569 nm; Cy3-insulin: excitation wavelength was 400 nm, emission wavelength was 540 nm). The dosage of released drugs was derived by comparing the measured fluorescence intensity with the standard curve.

### In vitro electric performance and ex vivo skin penetration test

The precleaned pig ear skin or mouse back skin (female C57BL6 mice) from fresh body was used. We removed the hair of skin by electric clippers and sanitized them using 75% alcohol. The Cy3-loaded MNs/Cy3-insulin-loaded MNs and blank MNs were attached to the anode electrode and cathode electrode of the device, respectively, using compont adhesive. MNs on the device were inserted inside the skin and waited for 5 min until MNs were fully swelled before turning on the power. The electrical properties (*n* = 3) under the duration of 0, 2, 5, 8, 10, 15, and 20 min and the current of 0, 0.1, 0.2, 0.3, 0.4, 0.5, 0.6, 0.8, 1, 2, 3, and 4 mA were calculated based on the electrical resistance measured by a DAQ multimeter (Keithley 6510, sampling rate: 60 kHz). The pulse pressing (0.5, 1, and 2 Hz) on the anode was achieved with a homemade oscillator. The constant current was powered by electrochemical station using the chronopotentiometry method.

In the skin penetration test, the fresh pig ear skin was used. MNs on the device were inserted into the skin for 5 min before turning on the power of electrochemical station. Group setting of Cy3-insulin delivery (*n* = 3): 0 mA for 10 min, 0 mA for 20 min, 0.5 mA for 10 min, 1 mA for 10 min, and 1 mA for 20 min. During the EF-controlled drug release, the pressing on the anode was constant to stabilize the output signal. Group setting of Cy3 delivery (*n* = 3): 0 mA for 5 min, 0.3 mA for 5 min, and 0.6 mA for 5 min. After EF-controlled drug release, the skin samples were embedded into tissue freezing medium and then sliced to 10-μm thickness using a cryostat (Leica CM3050S) for fluorescence imaging (excitation wavelength: 550 nm). The quantification of fluorescence intensity was calculated using ImageJ.

### In vivo insulin delivery experiments

The animal study protocol was approved by the Health Technology and Advisory Division, Department of Health, Hong Kong. Healthy adult mice (female C57BL6 mice; 6 to 8 weeks of age) were used. The mice were anesthetized with an anesthesia machine (R500IE, RWD). The device was applied onto the precleaned skin on the mouse back for 5 min until MNs were fully swelled. Then, the device was connected to flexible battery (4.35 V, 1,500 mAh). Multimeter was used to check whether the current was stable when the device was pressed by the finger. There were 5 mice in each of these groups: negative control (blank group, assembled with one pair of blank MNs without being pressed), positive control (intraperitoneal injection of 0.02 U of insulin), MN-Passive [one pair of MN electrodes without being pressed (anode MN loaded with 10 IU of insulin and blank cathode MN)], MN-Electrify (one pair of MN electrodes with 10-min electric stimulation via pressing), and blank MN-Electrify (one pair of blank MNs with 10-min electric stimulation via pressing). In the time points of 0, 10, 20, 30, 40, 60, 70, 80, 90, 100, and 120 min, the tail vein blood was collected for the BGL detection by the FreeStyle Optium Neo meter.

In the periodic release experiment, 5 mice were given to each of these groups: blank (device with only 2 pairs of blank MN electrodes and no pressing), MN-Electrify [2 pairs of MN electrodes (each anode MN loaded with 10 IU of insulin and cathode MN is blank) with periodic pressing], and MN-Passive (2 pairs of MN electrodes without pressing). The procedure for device operation was the same as above. To simulate the daily diet, glucose gavage (dosage: 1.5 g/kg) was administrated to all groups every 4 h as marked in Fig. 4C. The glucose levels were monitored each hour with a glucose meter over 13 h. The tail vein blood (~25 μl for each point) was collected and stored at −80 °C for insulin concentration quantification with a Human Insulin ELISA kit.

### Statistical analysis

All analyses were conducted with a sample size of *n* ≥ 3. The data were analyzed using Excel and Origin 8.5. Descriptive statistics were expressed as mean values ± SE. Student’s *t* test was used for comparison between data points. The statistical differences were assumed to be reproducible when *P* < 0.05.

## Data Availability

Data will be made available on request.

## References

[1] Prausnitz MR. Engineering microneedle patches for vaccination and drug delivery to skin. Annu Rev Chem Biomol Eng. 2017;8(1):177–200. 28375775 10.1146/annurev-chembioeng-060816-101514

[2] He J, Zhang Y, Yu X, Xu C. Wearable patches for transdermal drug delivery. Acta Pharm Sin B. 2023;13(6):2298–2309. 37425057 10.1016/j.apsb.2023.05.009 PMC10326306

[3] Peng K, Vora LK, Domínguez-Robles J, Naser YA, Li M, Larrañeta E, Donnelly RF. Hydrogel-forming microneedles for rapid and efficient skin deposition of controlled release tip-implants. Mater Sci Eng C. 2021;127:112226. 10.1016/j.msec.2021.112226 34225871

[4] Ning X, Wiraja C, Chew WTS, Fan C, Xu C. Transdermal delivery of Chinese herbal medicine extract using dissolvable microneedles for hypertrophic scar treatment. Acta Pharm Sin B. 2021;11(9):2937–2944. 34589406 10.1016/j.apsb.2021.03.016 PMC8463281

[5] Wang M, Han Y, Yu X, Liang L, Chang H, Yeo DC, Wiraja C, Wee ML, Liu L, Liu X, et al. Upconversion nanoparticle powered microneedle patches for transdermal delivery of siRNA. Adv Healthc Mater. 2020;9(2):1900635. 10.1002/adhm.201900635 31788987

[6] Chang H, Chew SWT, Zheng M, Lio DCS, Wiraja C, Mei Y, Ning X, Cui M, Than A, Shi P, et al. Cryomicroneedles for transdermal cell delivery. Nat Biomed Eng. 2021;5(9):1008–1018. 33941895 10.1038/s41551-021-00720-1

[7] Jiang X, Zhang W, Terry R, Li W. The progress of fabrication designs of polymeric microneedles and related biomedical applications. BMEMat. 2023;1(4): Article e12044.

[8] Yu J, Wang J, Zhang Y, Chen G, Mao W, Ye Y, Kahkoska AR, Buse JB, Langer R, Gu Z. Glucose-responsive insulin patch for the regulation of blood glucose in mice and minipigs. Nat Biomed Eng. 2020;4(5):499–506. 32015407 10.1038/s41551-019-0508-y PMC7231631

[9] Yu J, Zhang Y, Ye Y, DiSanto R, Sun W, Ranson D, Ligler FS, Buse JB, Gu Z. Microneedle-array patches loaded with hypoxia-sensitive vesicles provide fast glucose-responsive insulin delivery. Proc Natl Acad Sci U S A. 2015;112(27):8260–8265. 26100900 10.1073/pnas.1505405112 PMC4500284

[10] Wang C, Zeng Y, Chen K-F, Lin J, Yuan Q, Jiang X, Wu G, Wang F, Jia YG, Li W. A self-monitoring microneedle patch for light-controlled synergistic treatment of melanoma. Bioact Mater. 2023;27:58–71. 37035421 10.1016/j.bioactmat.2023.03.016 PMC10074410

[11] Xu L, Yang Y, Mao Y, Li Z. Self-powerbility in electrical stimulation drug delivery system. Adv Mater Technol. 2022;7(2):2100055.

[12] Zhou Y, Jia X, Pang D, Jiang S, Zhu M, Lu G, Tian Y, Wang C, Chao D, Wallace G. An integrated Mg battery-powered iontophoresis patch for efficient and controllable transdermal drug delivery. Nat Commun. 2023;14(1):297. 36653362 10.1038/s41467-023-35990-7 PMC9849227

[13] Kusama S, Sato K, Matsui Y, Kimura N, Abe H, Yoshida S, Nishizawa M. Transdermal electroosmotic flow generated by a porous microneedle array patch. Nat Commun. 2021;12(1):658. 33510169 10.1038/s41467-021-20948-4 PMC7843990

[14] Yang Y, Xu L, Jiang D, Chen BZ, Luo R, Liu Z, Qu X, Wang C, Shan Y, Cui Y, et al. Self-powered controllable transdermal drug delivery system. Adv Funct Mater. 2021;31(36):2104092.

[15] Li J, Yang T, Huang D, Chen Y, Huang Y, Li Z. High-density microneedle array (HIDMA): An in vivo electroporation method for low-voltage gene delivery. Paper presented at: 2020 IEEE 33rd International Conference on Micro Electro Mechanical Systems (MEMS); 2020; Vancouver, Canada.

[16] Huang Y, Li H, Hu T, Li J, Yiu CK, Zhou J, Li J, Huang X, Yao K, Qiu X, et al. Implantable electronic medicine enabled by bioresorbable microneedles for wireless electrotherapy and drug delivery. Nano Lett. 2022;22(14):5944–5953. 35816764 10.1021/acs.nanolett.2c01997

[17] Amini-Nik S, Yousuf Y, Jeschke MG. Scar management in burn injuries using drug delivery and molecular signaling: Current treatments and future directions. Adv Drug Deliv Rev. 2018;123:135–154. 28757325 10.1016/j.addr.2017.07.017 PMC5742037

[18] Lyu S, Dong Z, Xu X, Bei HP, Yuen HY, James Cheung CW, Wong MS, He Y, Zhao X. Going below and beyond the surface: Microneedle structure, materials, drugs, fabrication, and applications for wound healing and tissue regeneration. Bioact Mater. 2023;27:303–326. 37122902 10.1016/j.bioactmat.2023.04.003 PMC10140753

[19] Tehrani F, Teymourian H, Wuerstle B, Kavner J, Patel R, Furmidge A, Aghavali R, Hosseini-Toudeshki H, Brown C, Zhang F, et al. An integrated wearable microneedle array for the continuous monitoring of multiple biomarkers in interstitial fluid. Nat Biomed Eng. 2022;6(11):1214–1224. 35534575 10.1038/s41551-022-00887-1

[20] Lee W, Jeong S-H, Lim Y-W, Lee H, Kang J, Lee H, Lee I, Han H-S, Kobayashi S, Tanaka M, et al. Conformable microneedle pH sensors via the integration of two different siloxane polymers for mapping peripheral artery disease. Sci Adv. 2021;7(48):eabi6290. 34826244 10.1126/sciadv.abi6290 PMC8626065

[21] Ren L, Xu S, Gao J, Lin Z, Chen Z, Liu B, Liang L, Jiang L. Fabrication of flexible microneedle array electrodes for wearable bio-signal recording. Sensors. 2018;18(4):1191. 29652835 10.3390/s18041191 PMC5948552

[22] Lee H, Choi TK, Lee YB, Cho HR, Ghaffari R, Wang L, Choi HJ, Chung TD, Lu N, Hyeon T, et al. A graphene-based electrochemical device with thermoresponsive microneedles for diabetes monitoring and therapy. Nat Nanotechnol. 2016;11(6):566–572. 26999482 10.1038/nnano.2016.38

[23] Suarez-Roca H, Klinger RY, Podgoreanu MV, Ji RR, Sigurdsson MI, Waldron N, Mathew JP, Maixner W. Contribution of baroreceptor function to pain perception and perioperative outcomes. Anesthesiology. 2019;130(4):634 650. 30418212 10.1097/ALN.0000000000002510 PMC6417948

[24] Chang H, Zheng M, Yu X, Than A, Seeni RZ, Kang R, Tian J, Khanh DP, Liu L, Chen P, et al. A swellable microneedle patch to rapidly extract skin interstitial fluid for timely metabolic analysis. Adv Mater. 2017;29(37):1702243. 10.1002/adma.201702243 28714117

[25] Zhou Y, Wang G, Liu J, Du Y, Wang L, Wang X. Application of COMPONT medical adhesive glue for tension-reduced duraplasty in decompressive craniotomy. Med Sci Monit. 2016;22:3689–3693. 27752035 10.12659/MSM.896982 PMC5072381

[26] Summerfield A, Meurens F, Ricklin ME. The immunology of the porcine skin and its value as a model for human skin. Mol Immunol. 2015;66(1):14–21. 25466611 10.1016/j.molimm.2014.10.023

[27] Jiang Y, Trotsyuk AA, Niu S, Henn D, Chen K, Shih C-C, Larson MR, Mermin-Bunnell AM, Mittal S, Lai JC, et al. Wireless, closed-loop, smart bandage with integrated sensors and stimulators for advanced wound care and accelerated healing. Nat Biotechnol. 2023;41(5):652–662. 36424488 10.1038/s41587-022-01528-3

[28] J. Li, T. Yang, D. Huang, Y. Chen, Y. Huang, Z. Li, High-density microneedle array (HIDMA): An in vivo electroporation method for low-voltage gene delivery. Paper presented at: 2020 IEEE 33rd International Conference on Micro Electro Mechanical Systems (MEMS); 2020; Vancouver, Canada.

[29] Sadeqi A, Kiaee G, Zeng W, Rezaei Nejad H, Sonkusale S. Hard polymeric porous microneedles on stretchable substrate for transdermal drug delivery. Sci Rep. 2022;12(1):1853. 35115643 10.1038/s41598-022-05912-6 PMC8813900

[30] Gao Y, Zhang B, Liu Y, Yao K, Huang X, Li J, Wong TH, Huang Y, Li J, Zhou J, et al. Mechanoreceptor inspired electronic skin for multi-modal tactile information decoding. Adv Mater Technol. 2023;8(1):2200759.

[31] Yao K, Zhou J, Huang Q, Wu M, Yiu CK, Li J, Huang X, Li D, Su J, Hou S, et al. Encoding of tactile information in hand via skin-integrated wireless haptic interface. Nat Mach Intell. 2022;4(10):893–903.

[32] Yiu C, Liu Y, Zhang C, Zhou J, Jia H, Wong TH, Huang X, Li J, Yao K, Yau MK, et al. Soft, stretchable, wireless intelligent three-lead electrocardiograph monitors with feedback functions for warning of potential heart attack. SmartMat. 2022;3(4):668–684.

[33] Liang G, Ruan Z, Liu Z, Li H, Wang Z, Tang Z, Mo F, Yang Q, Ma L, Wang D, et al. Toward multifunctional and wearable smart skins with energy-harvesting, touch-sensing, and exteroception-visualizing capabilities by an all-polymer design. Adv Electron Mater. 2019;5(10):1900553.

[34] Yang Q, Chen A, Li C, Zou G, Li H, Zhi C. Categorizing wearable batteries: Unidirectional and omnidirectional deformable batteries. Matter. 2021;4(10):3146–3160.

[35] Seeni RZ, Zheng M, Lio DCS, Wiraja C, Mohd Yusoff MFB, Koh WTY, Liu Y, Goh BT, Xu C. Targeted delivery of anesthetic agents to bone tissues using conductive microneedles enhanced iontophoresis for painless dental anesthesia. Adv Funct Mater. 2021;31(47):2105686.

[36] Hu T, Zhang Z, Xu C. Transdermal delivery of dextran using conductive microneedles assisted by iontophoresis. J Mater Chem B. 2022;10(39):8075–8081. 36124549 10.1039/d2tb01049f

[37] Fish RM, Geddes LA. Conduction of electrical current to and through the human body: A review. Eplasty. 2009;9:e44. 19907637 PMC2763825

[38] Xu X, Zhang H, Yan Y, Wang J, Guo L. Effects of electrical stimulation on skin surface. Acta Mech Sin. 2021;37(12):1843–1871. 33584001 10.1007/s10409-020-01026-2 PMC7866966

