# Supplementary material for: Baroreceptor-Inspired Microneedle Skin Patch for Pressure-Controlled Drug Release
Source: BME Front. 2024 Jun 28;5:0044. doi: 10.34133/bmef.0044 (PMC11210744; doi:10.34133/bmef.0044)
Supplement: Supplementary 1 — Figs. S1 to S15 Table S1 [file bmef.0044.f1.zip › Support information_revised.docx]

**Baroreceptor-inspired microneedle skin patch for pressure-controlled drug release**

Jiahui He^1^, Mengjia Zheng^1^, Tianli Hu^1^, Ya Huang^1,3^, Jingyou Su^1^, Chunyi Zhi^2^, Xinge Yu^1,3*^, Chenjie Xu^1*^

1. Department of Biomedical Engineering, City University of Hong Kong, Tat Chee Ave, Kowloon, Hong Kong SAR, China

2. Department of Materials Science and Engineering, City University of Hong Kong, Tat Chee Ave, Kowloon, Hong Kong SAR, China

3. Hong Kong Centre for Cerebro-Cardiovascular Health Engineering, Hong Kong Science Park, New Territories, Hong Kong SAR, China

*Corresponding author. Email: [chenjie.xu@cityu.edu.hk](mailto:chenjie.xu@cityu.edu.hk); xingeyu@cityu.edu.hk

**Supplementary information**

Table S1. The corrosion current and corrosion voltage of uncoated copper, carbon-coated copper and Ni&Au coated copper.

|  | **Cu** | **Carbon** | **Ni&Au** |
| --- | --- | --- | --- |
| Icorr | 0.132 µA | 0.098 µA | 0.078 µA |
| Vcorr | -207 mV | -211 mV | -79 mV |


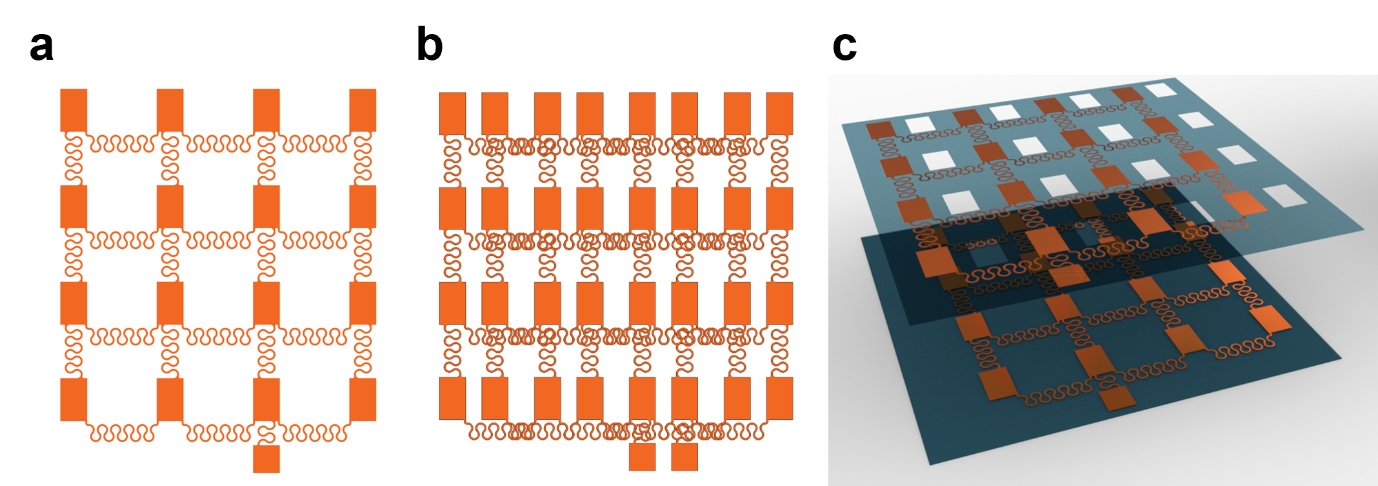


Figure S1. The alignment of the double-layers electrode array: (a) The single electrode pattern; (b) The aligning of anode and cathode patterns; (c) The alignment of the cathode (up) and anode (down).


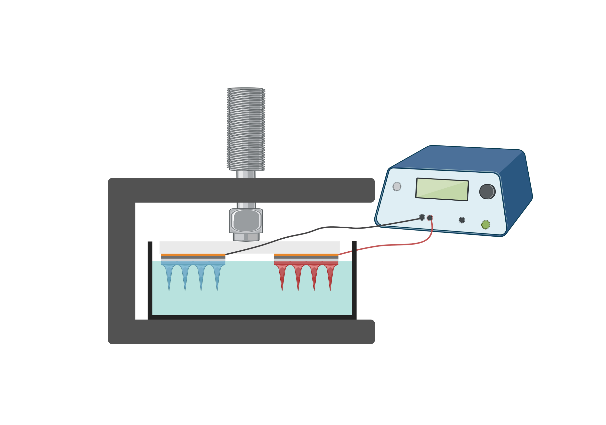


Figure S2. Illustration of *in-vitro* drug release test.


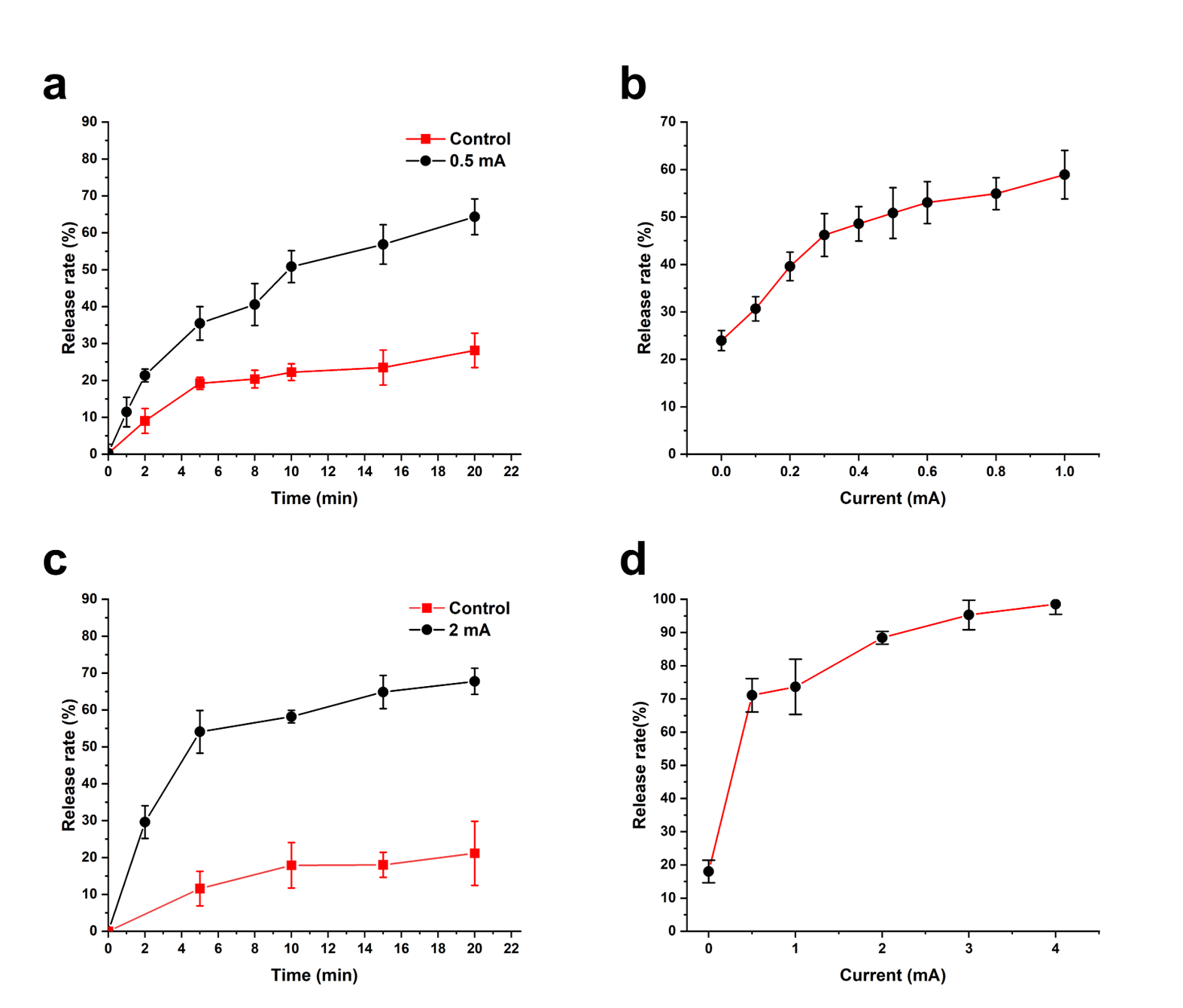


Figure S3. The EF induced drug release in the hydrogel model: (a) The release of Cy3 from MeHA MNs over time with or without the constant current of 0.5 mA; (b) The release of Cy3 from MeHA MNs as a function of current with the same powering time of 15 min; (c) The release of Cy3-insulin from MeHA MNs over time with or without the constant current of 2 mA; (d) The release of Cy3-insulin from MeHA MNs as a function of current with the same powering time of 30 min.


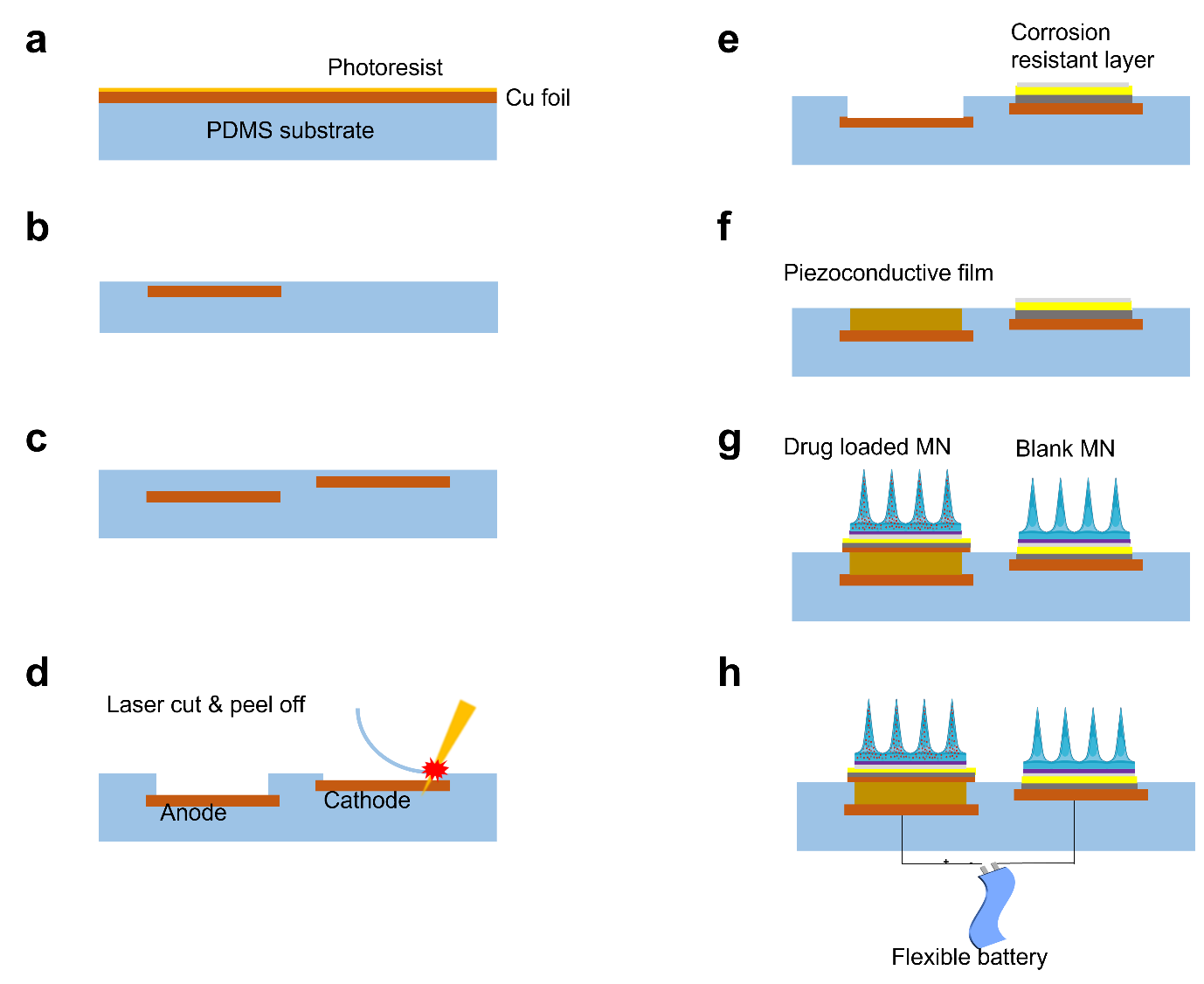


Figure S4. The fabrication process of MN skin patch: (a)~(d) Double-layer electrode array fabrication by photolithography and laser cutting; (e) Anti-corrosion layer deposited by electroplated nickel gold, and Ag/AgCl paste coating in sequence; (f) Piezoconductive film assembling; (g) MNs assembling by adhesive glue; (h) Flexible battery connection.


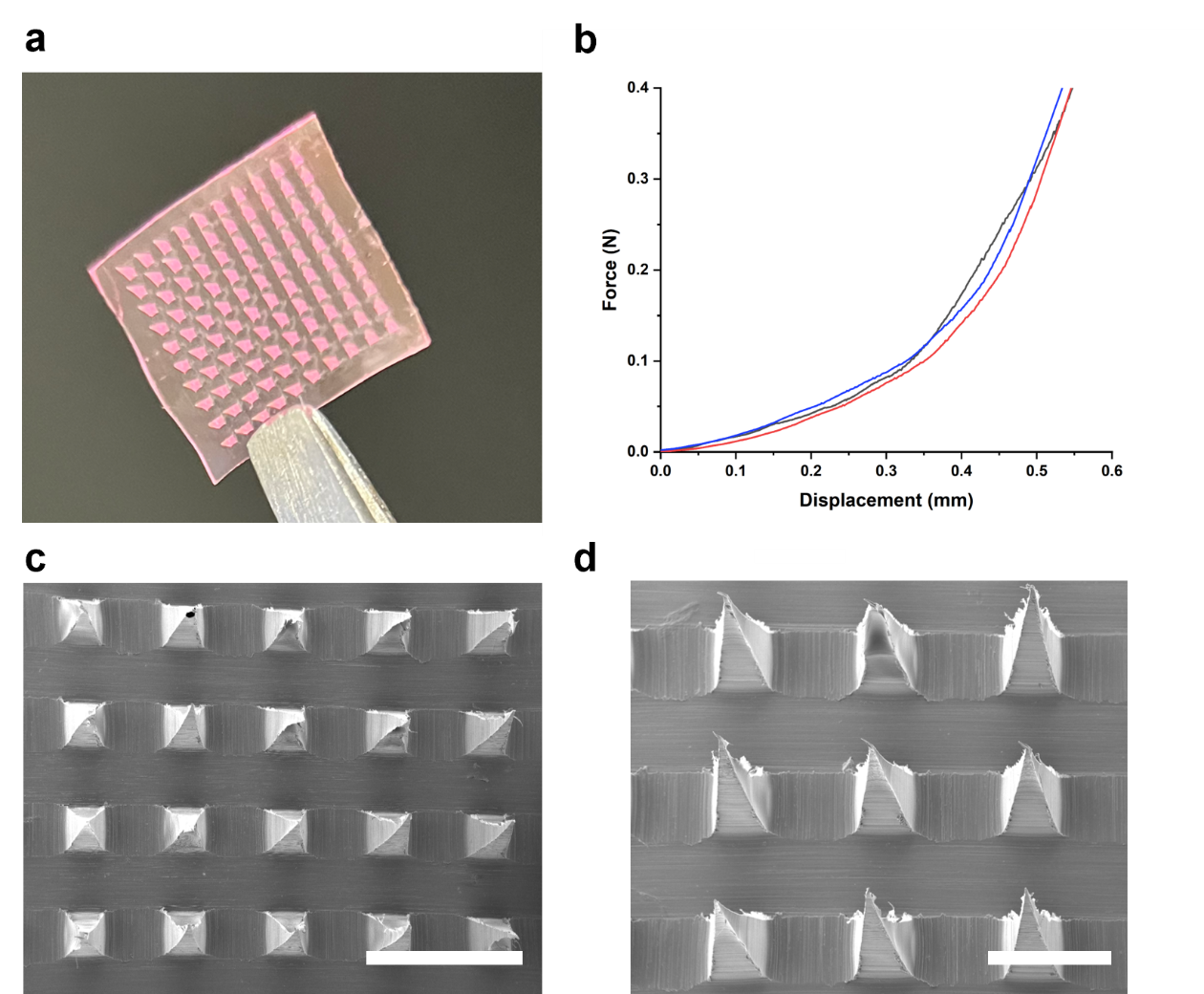


Figure S5. Characterization of the MeHA MN: (a) The optical image of as-prepared MeHA MN patch (Cy3-insulin loaded); (b) the mechanical compression tests of MeHA MNs (x3); (c) The SEM image of MeHA MNs (scare bar: 1 mm); (d) the zoom-in SEM image of MNs (scare bar: 500 µm).


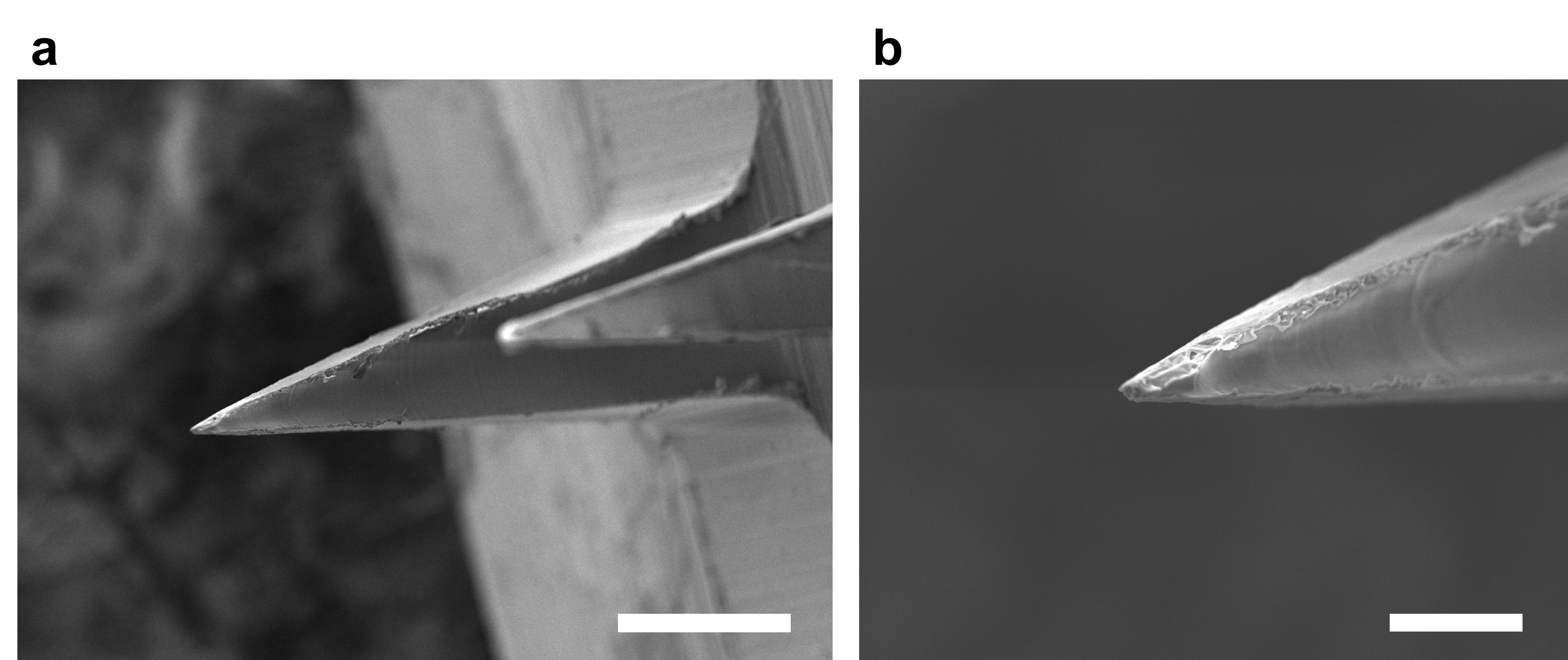


Figure S6. The SEM image of MN tips: (a) A single needle (scale bar: 100 µm) and (b) the magnified image of the tip. (scale bar: 50 µm)


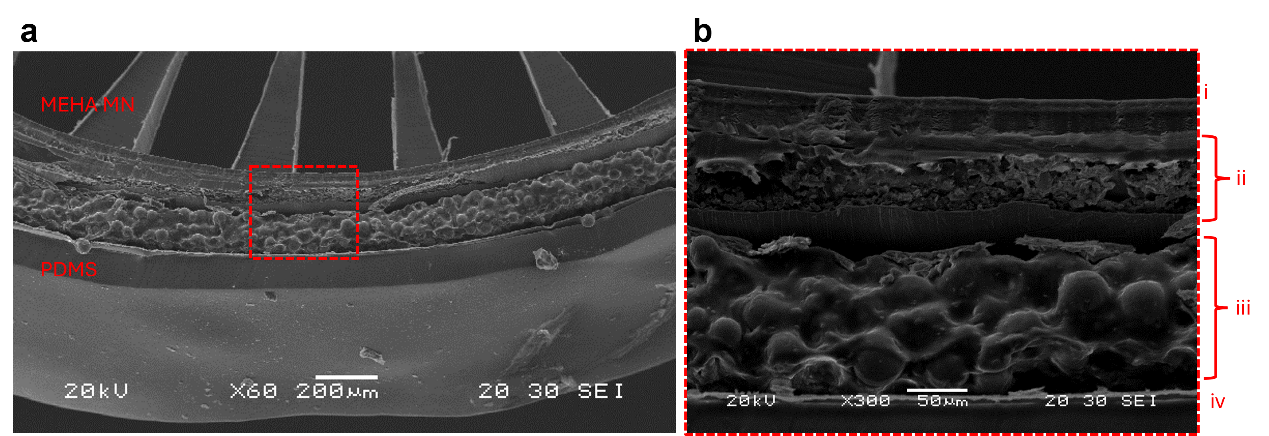


Figure S7. The sectional view of an anode MN unit: (a) the sectional structure of an anode MN unit; (b) the enlarged image presenting the elemental composition among different layers: (i) MEHA backpatch, (ii) anti-corrosion coated copper layer, (iii) piezo conductive film and (iv) copper electrode.


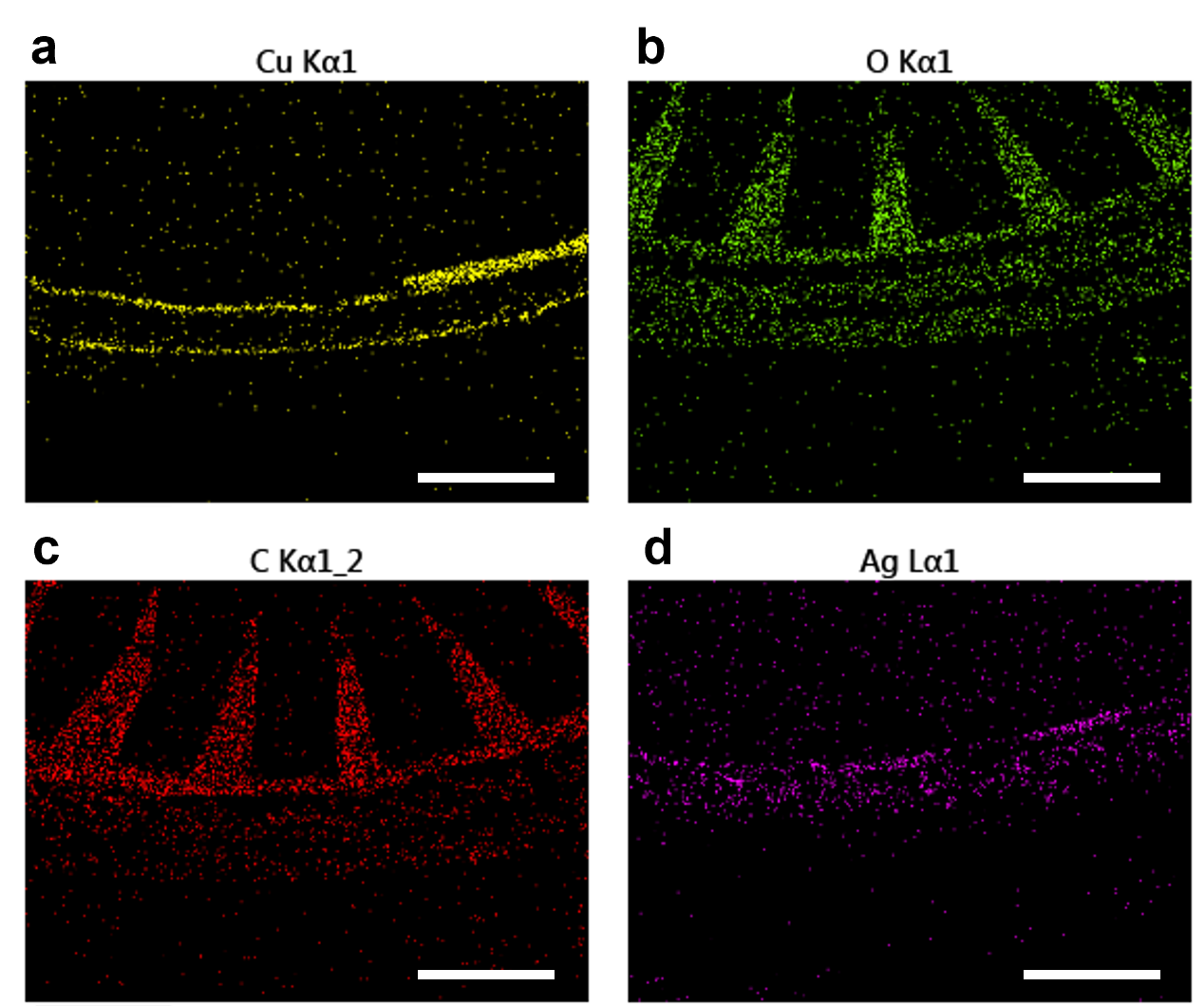


Figure S8. The EDS analysis of an anode MN unit: (a) Cu double layers represented the double-electrode patterns; (b, c) represented the MEHA MN and piezo conductive film; (d) represented the Ag/AgCl coating and piezo conductive film (scale bar: 500 µm).


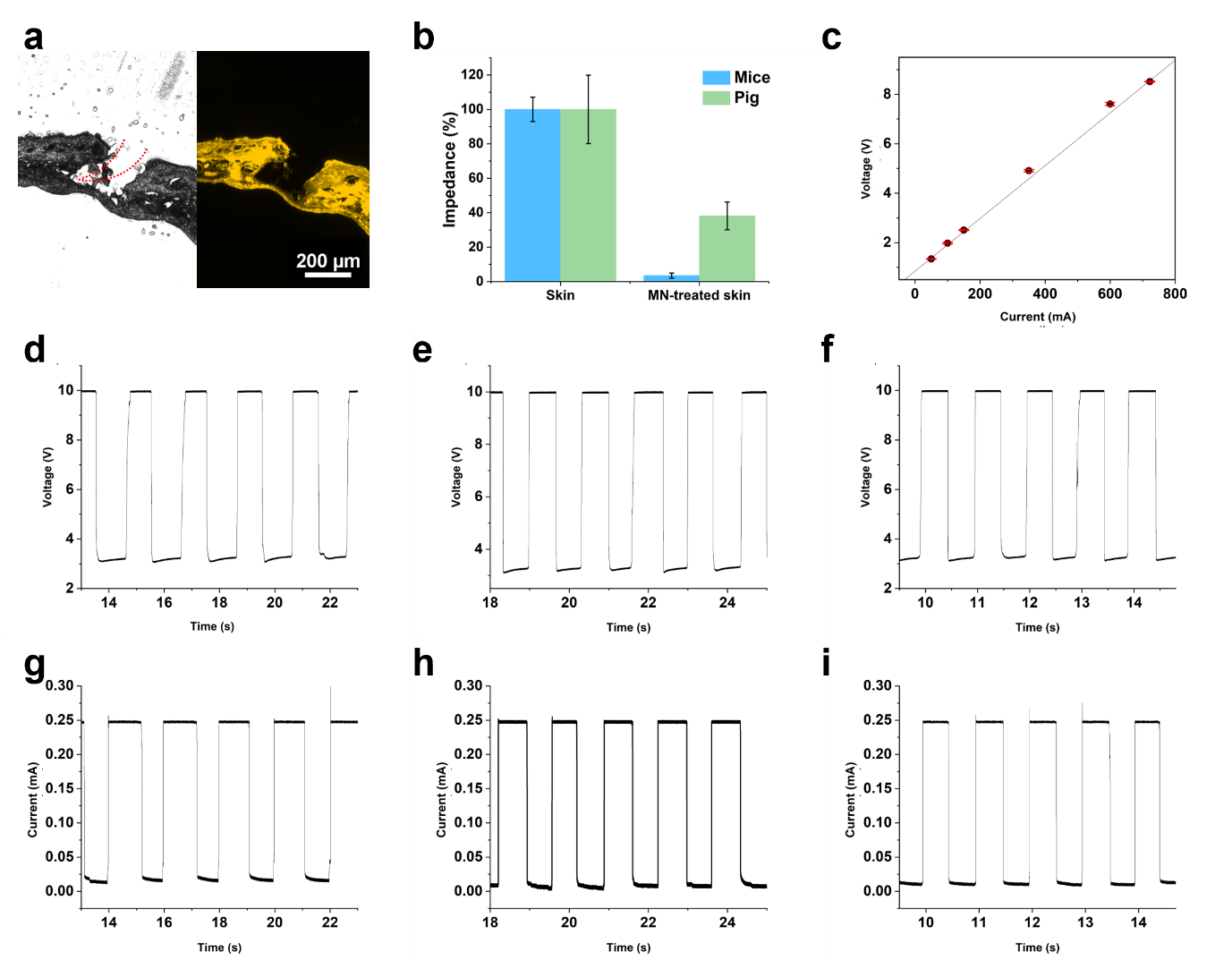


Figure S9. Electrical response of the MN device array on the skin: (a) The optical image (left) and Cy3 distribution (right) in the sliced section of mice skin; (b) The electrical impedance of pig and mice skin with/without MN device treatment; (c) Load voltage as a function of constant current; (d-f) The voltage change and (g-i) the current change of the MN device upon the perioidic pressing at the frequencies of 0.5 Hz, 1 Hz, and 2 Hz.


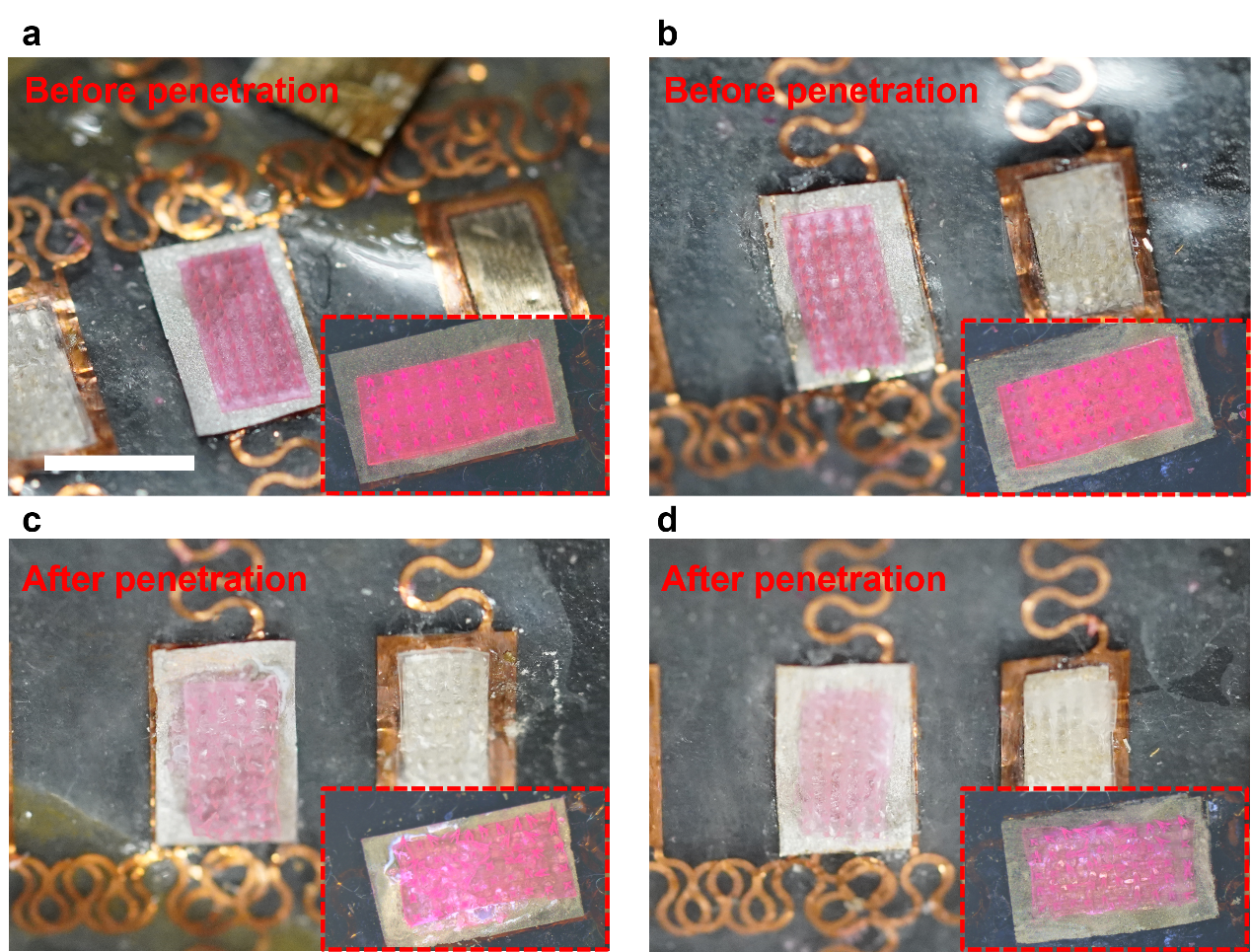


Figure S10. The optical images of MN device: (a) before and (c) after the penetration on pig skin; (b) before and (d) after the penetration on mice skin (scale bar: 5 mm).


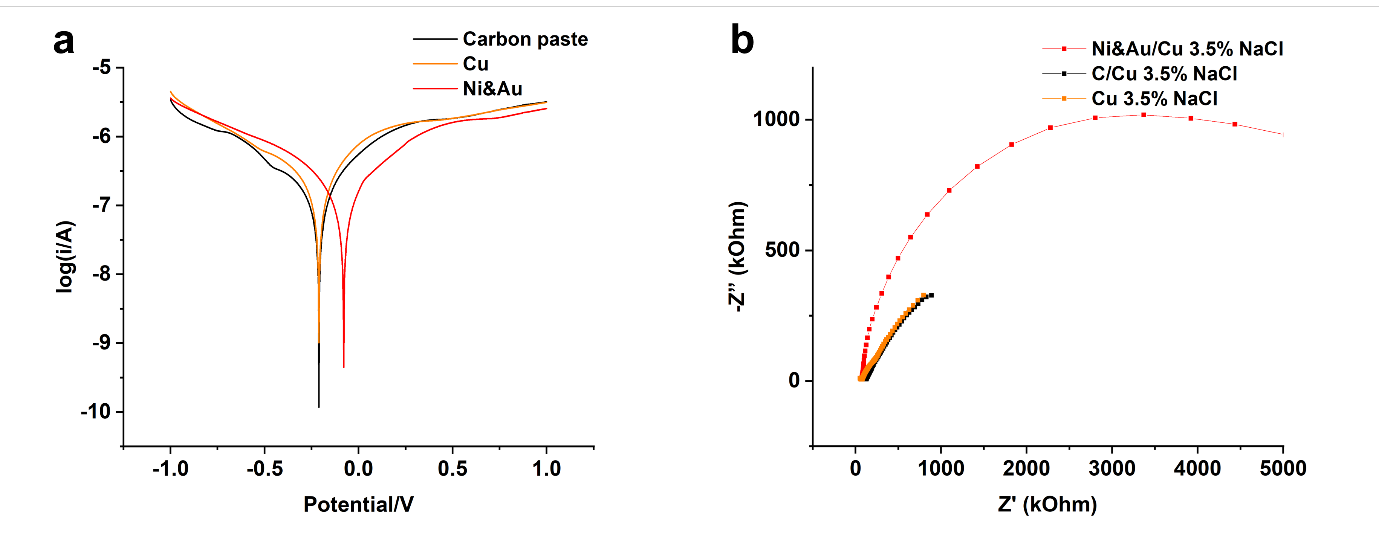


Figure S11. The anti-corrsion test. (a) The Tafel plots and (b) Nyquist plots for uncoated copper electrode, carbon-coated, Ni/Au-coated electrodes measured in the 3.5 wt % NaCl aqueous solution (potentiodynamic analysis).


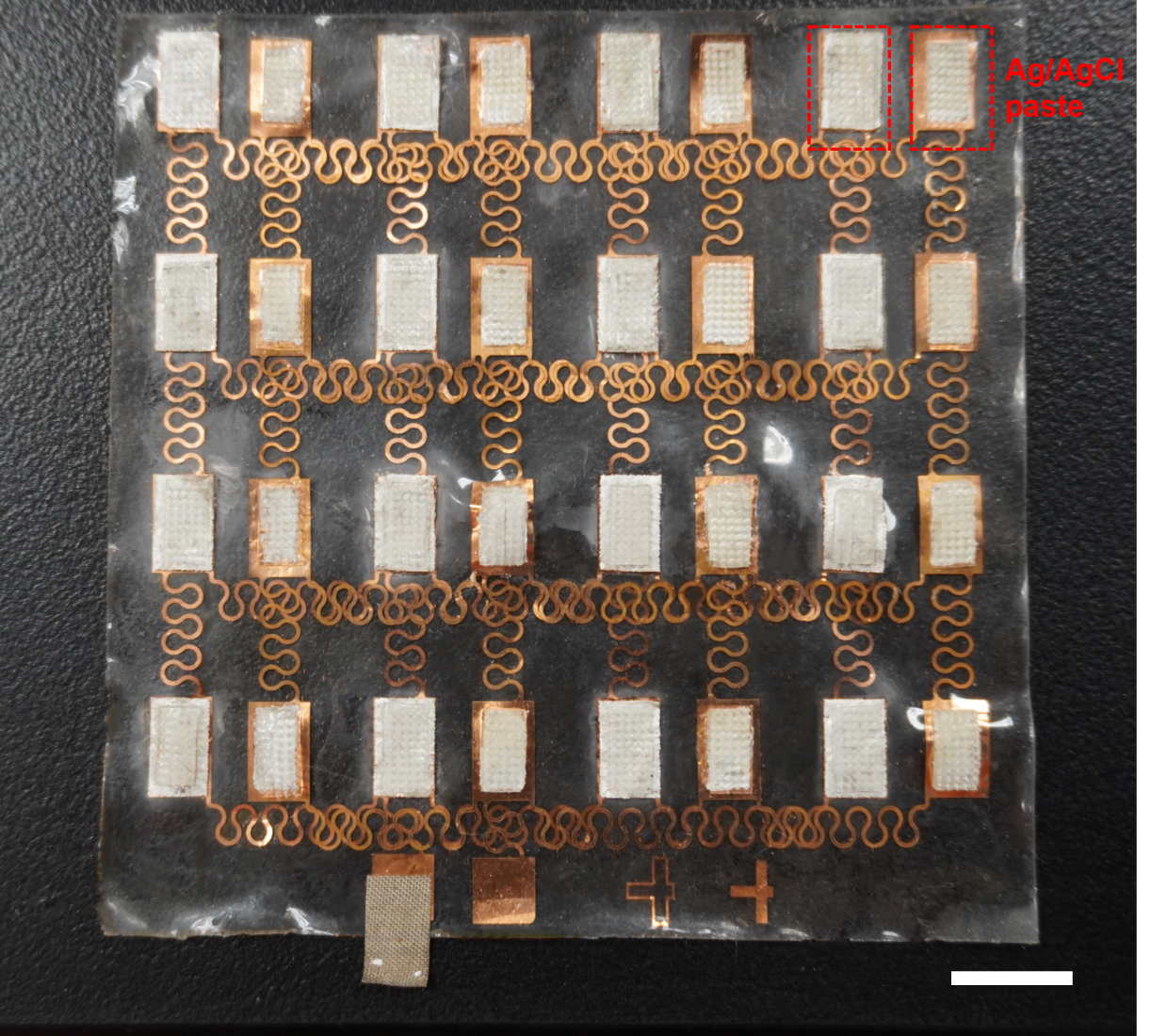


Figure S12. The MN device with the Ag/AgCl paste coating (scare bar: 1 cm)


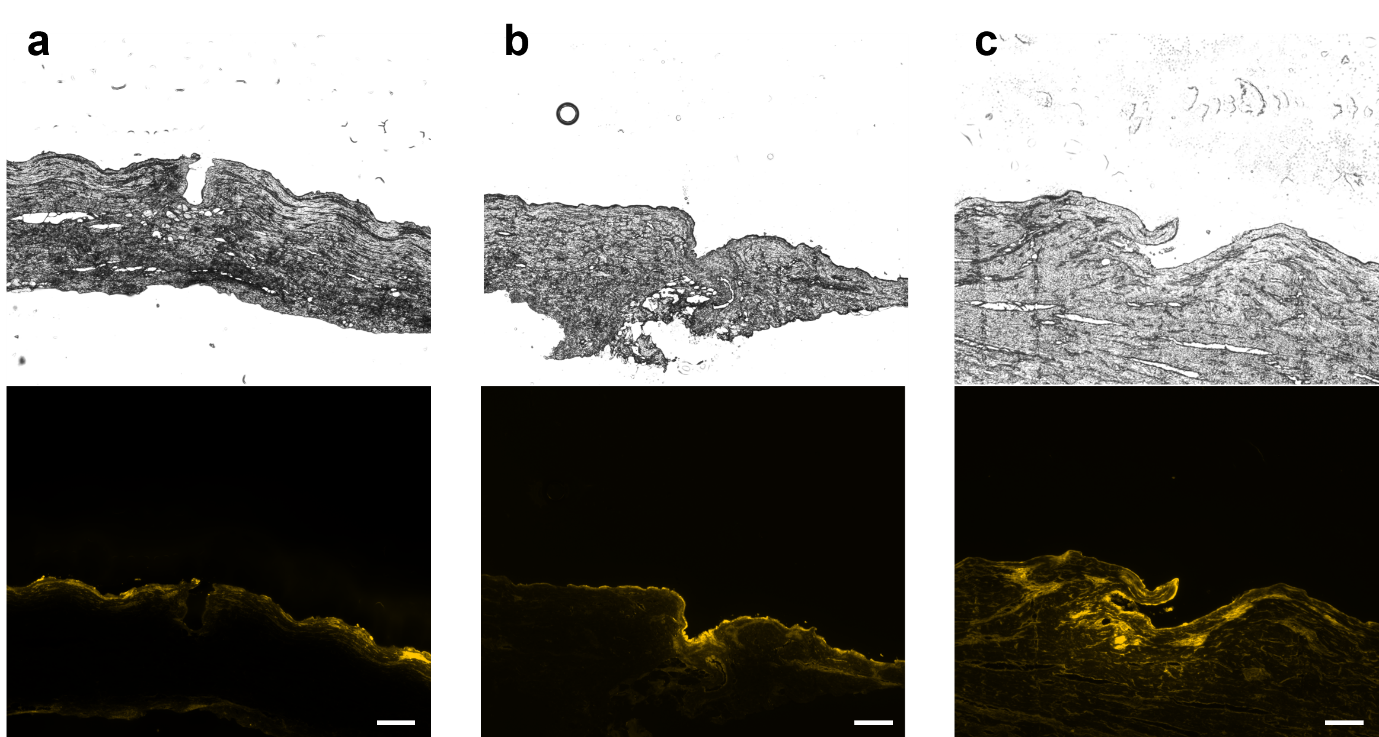


Figure S13. Pressing induced Cy3 release in pig ear skin after being applied with (b) 0 mA for 5 min; (c) 0.3 mA for 5 min; (d) 0.6 mA for 5 min. (scale bar: 200 µm)


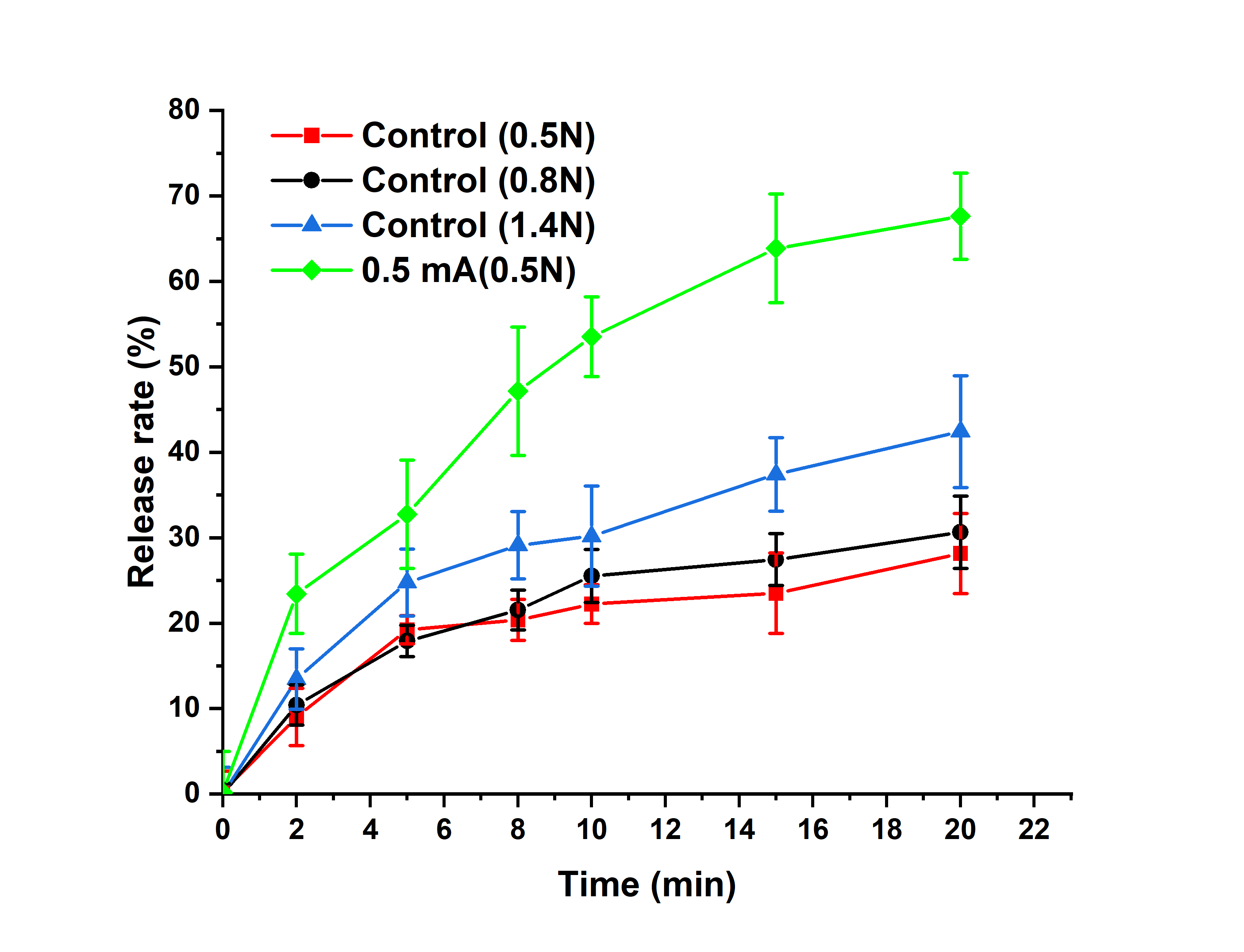


Figure S14. The drug release profiles from the devices applied with different forces with or without current.


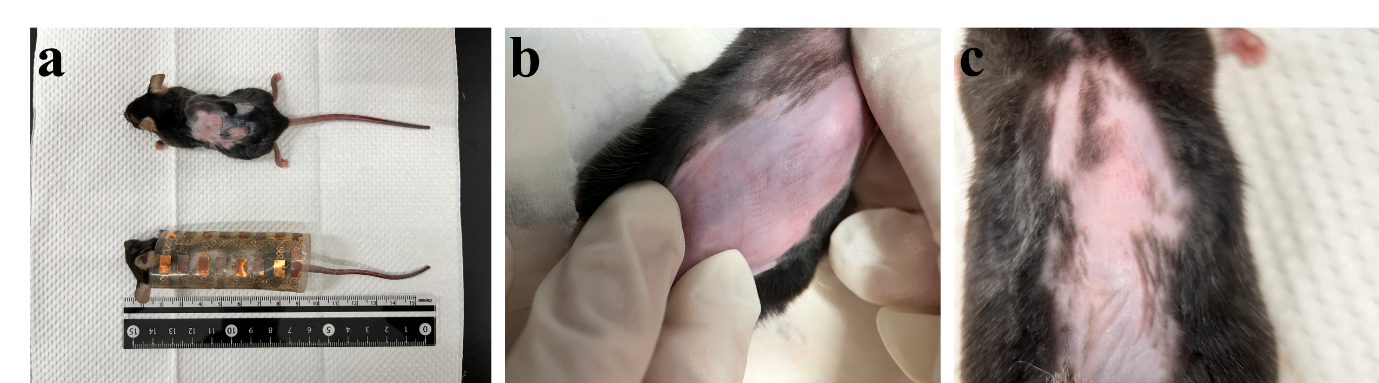


Figure S15. Skin recovery after MN application: (a) The negative control group (up) and device group (down); The mouse skin image (b) right after MN removal and (c) 5 min after MN removal.
