# Supplementary figures and images for: Baroreceptor-Inspired Microneedle Skin Patch for Pressure-Controlled Drug Release
Source: BME Front. 2024 Jun 28;5:0044. doi: 10.34133/bmef.0044 (PMC11210744; doi:10.34133/bmef.0044)

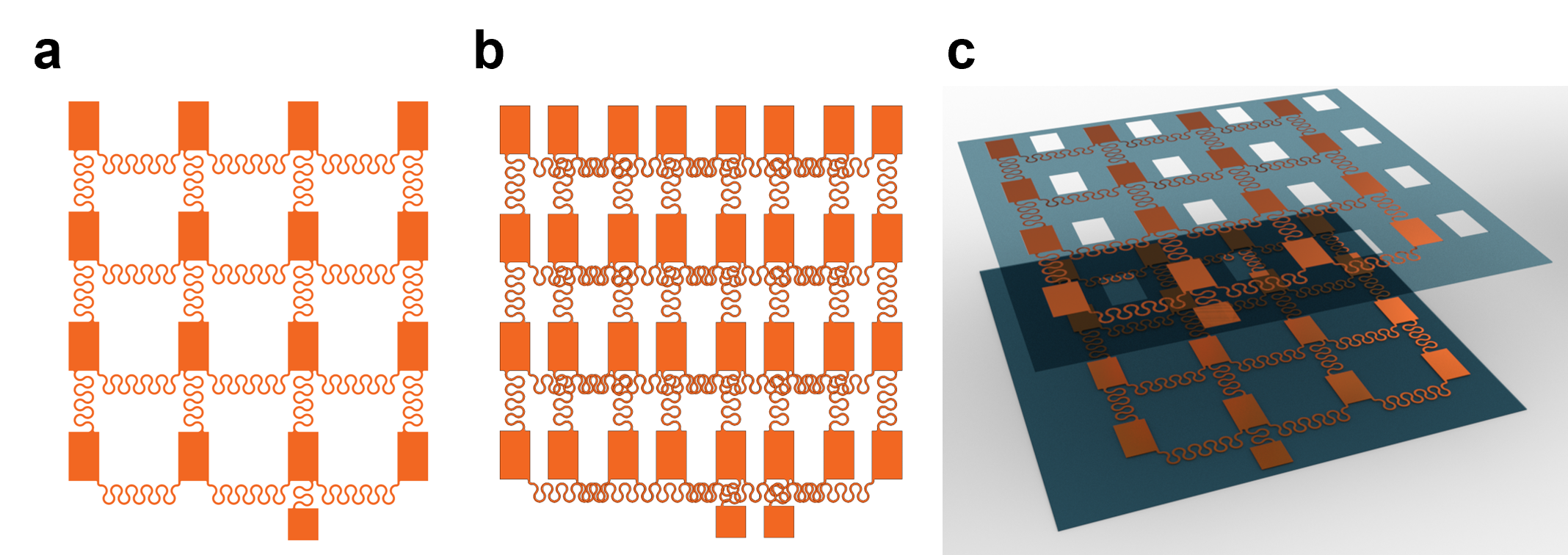

Supplement: Supplementary 1 — Figs. S1 to S15 Table S1 [file bmef.0044.f1.zip › FS1.png]

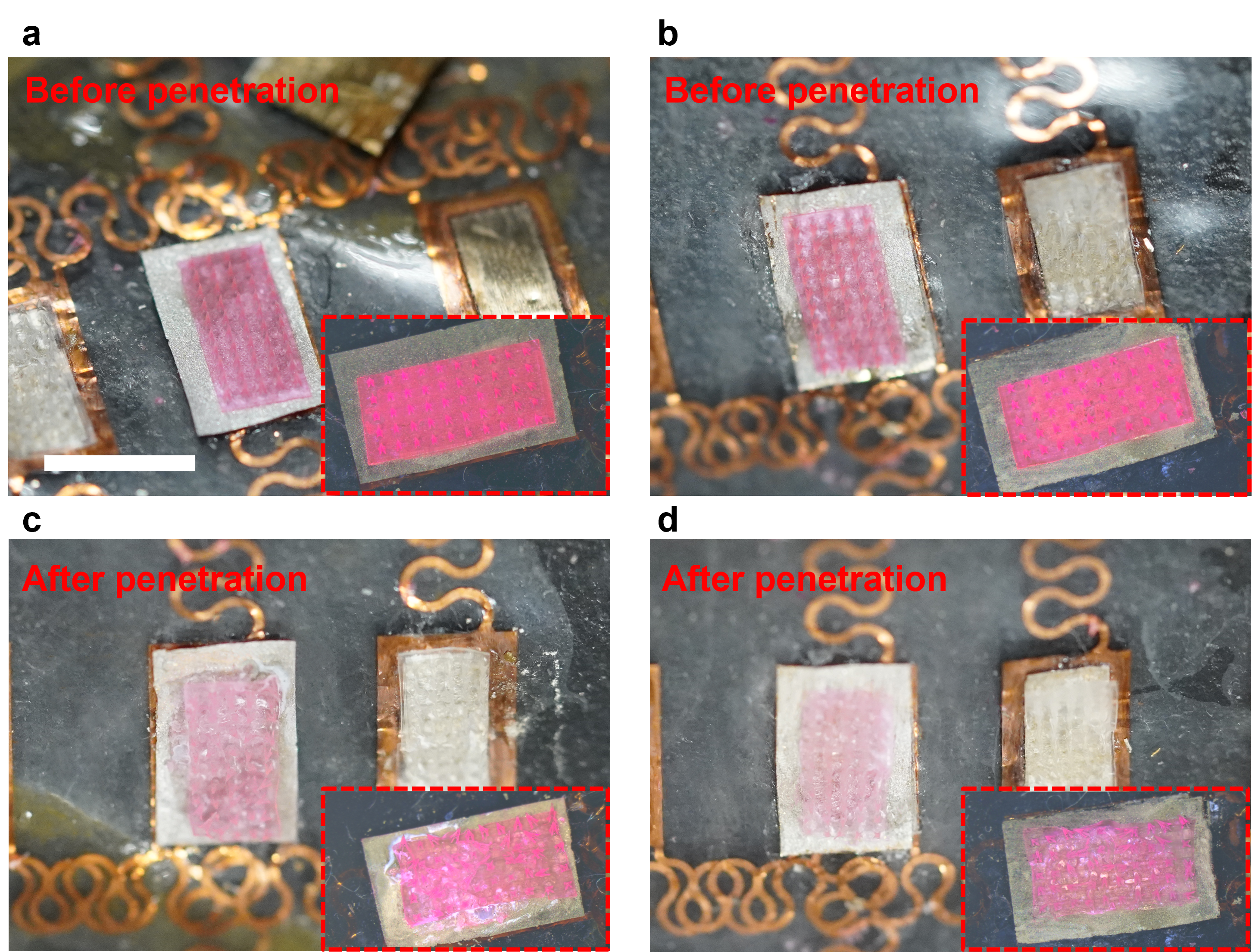

Supplement: Supplementary 1 — Figs. S1 to S15 Table S1 [file bmef.0044.f1.zip › FS10 .png]

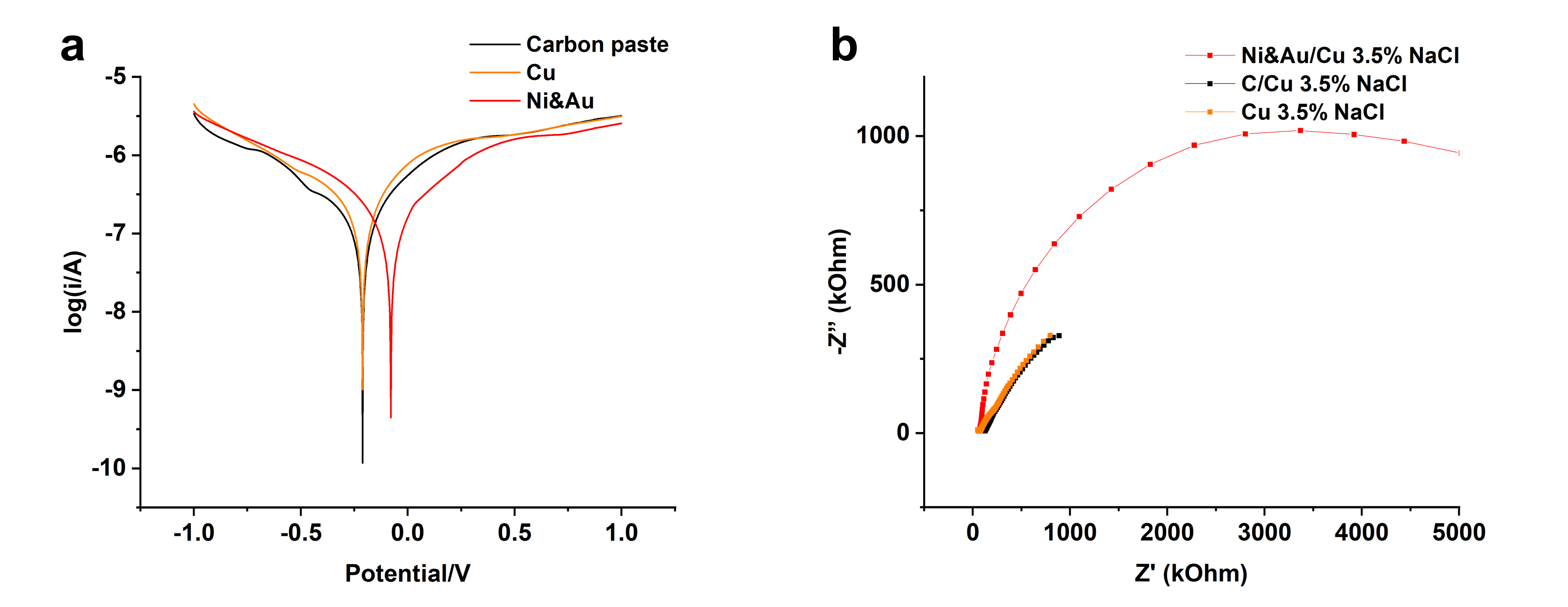

Supplement: Supplementary 1 — Figs. S1 to S15 Table S1 [file bmef.0044.f1.zip › FS11 .png]

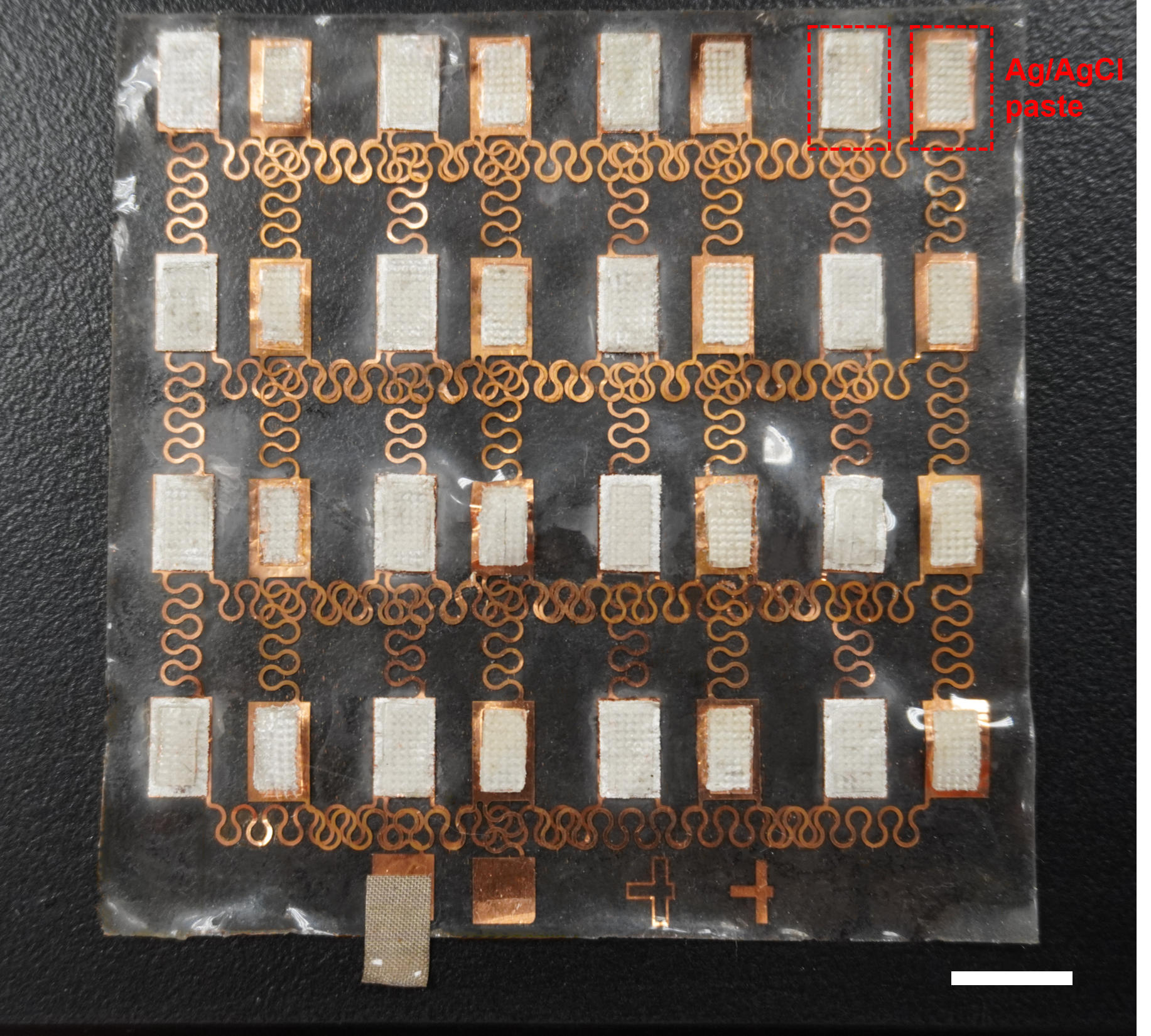

Supplement: Supplementary 1 — Figs. S1 to S15 Table S1 [file bmef.0044.f1.zip › FS12.png]

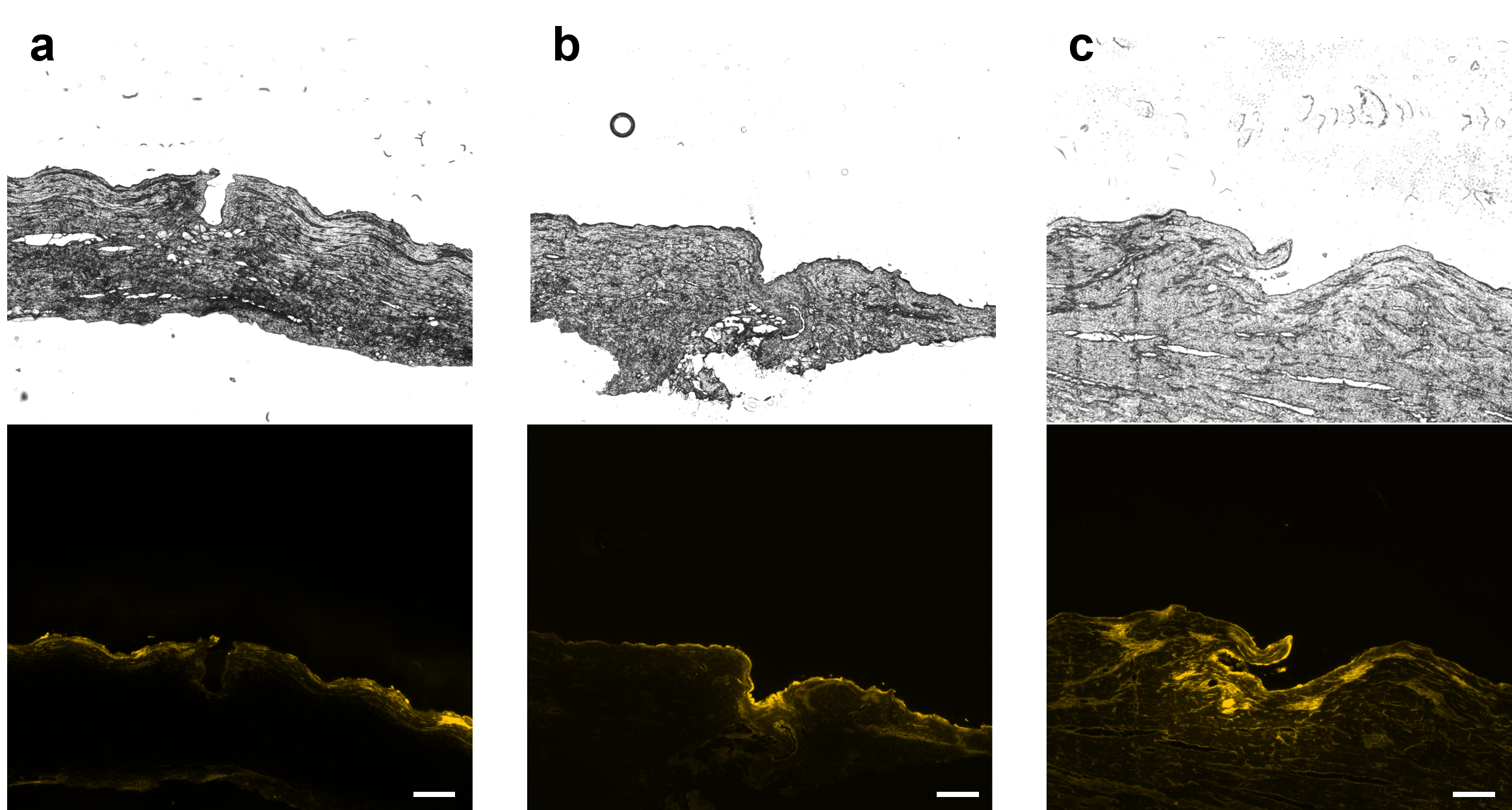

Supplement: Supplementary 1 — Figs. S1 to S15 Table S1 [file bmef.0044.f1.zip › FS13 .png]

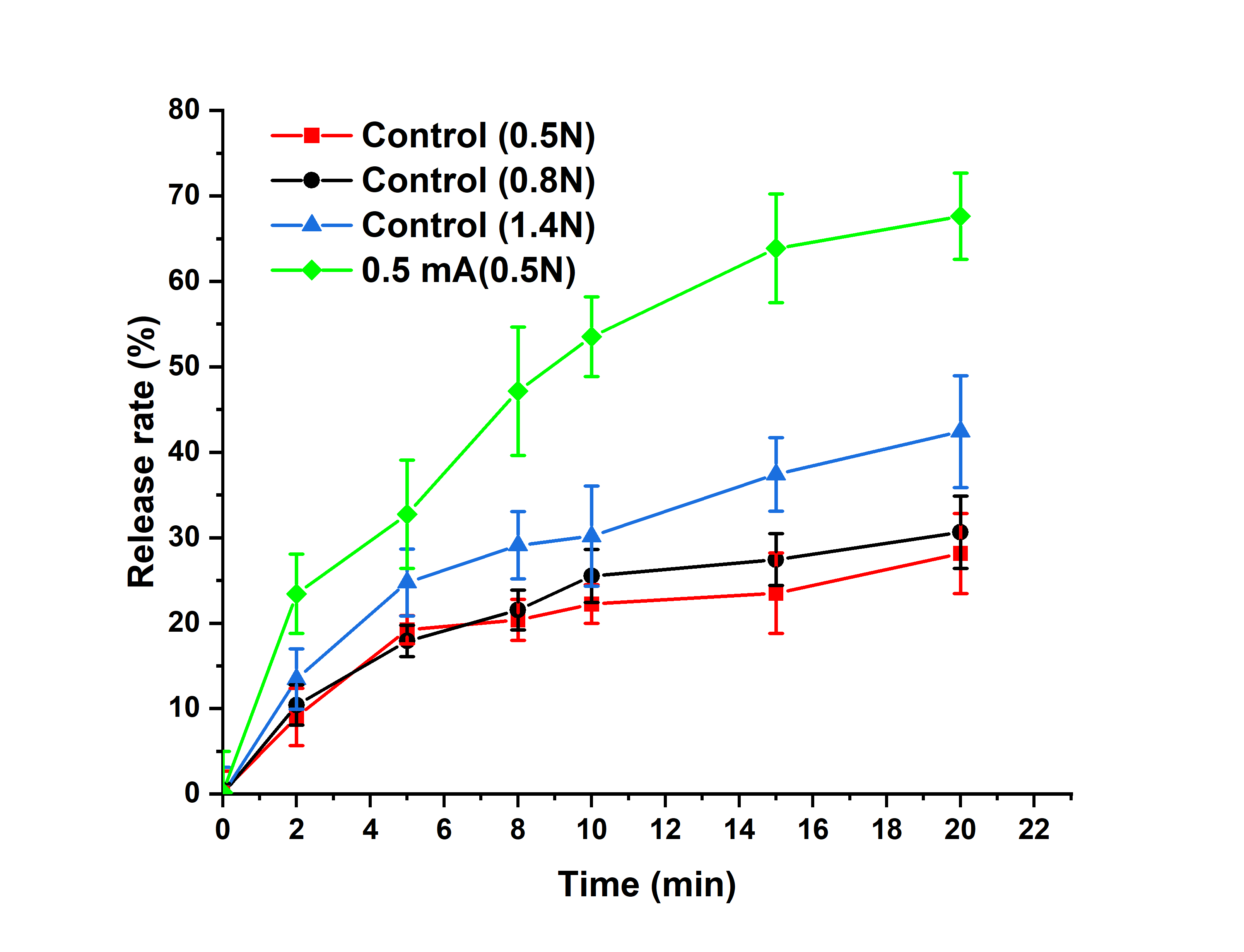

Supplement: Supplementary 1 — Figs. S1 to S15 Table S1 [file bmef.0044.f1.zip › FS14 .png]

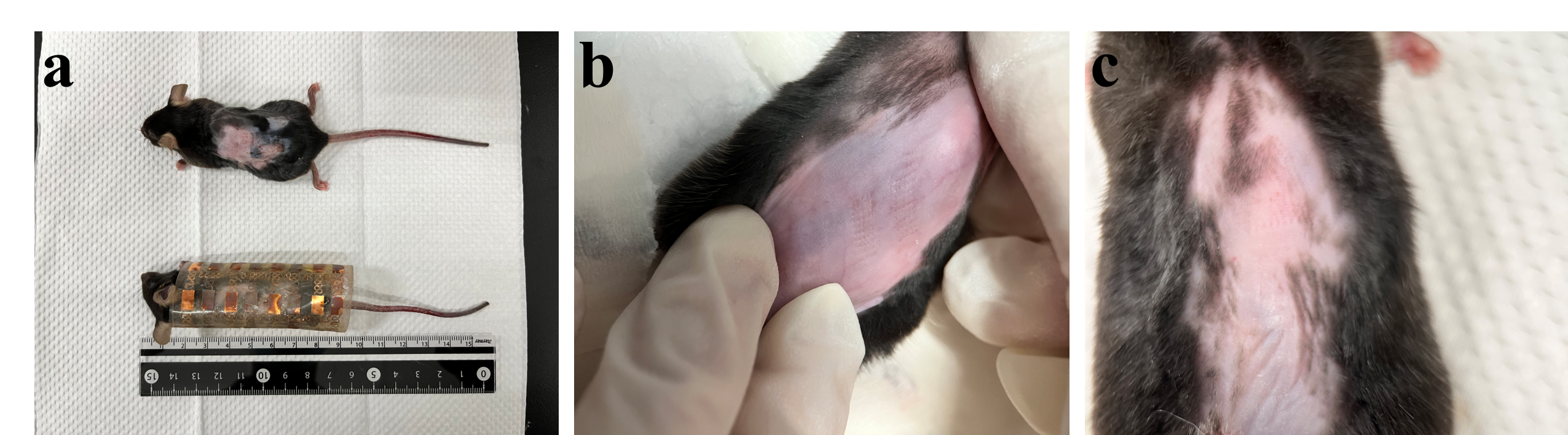

Supplement: Supplementary 1 — Figs. S1 to S15 Table S1 [file bmef.0044.f1.zip › FS15 .png]

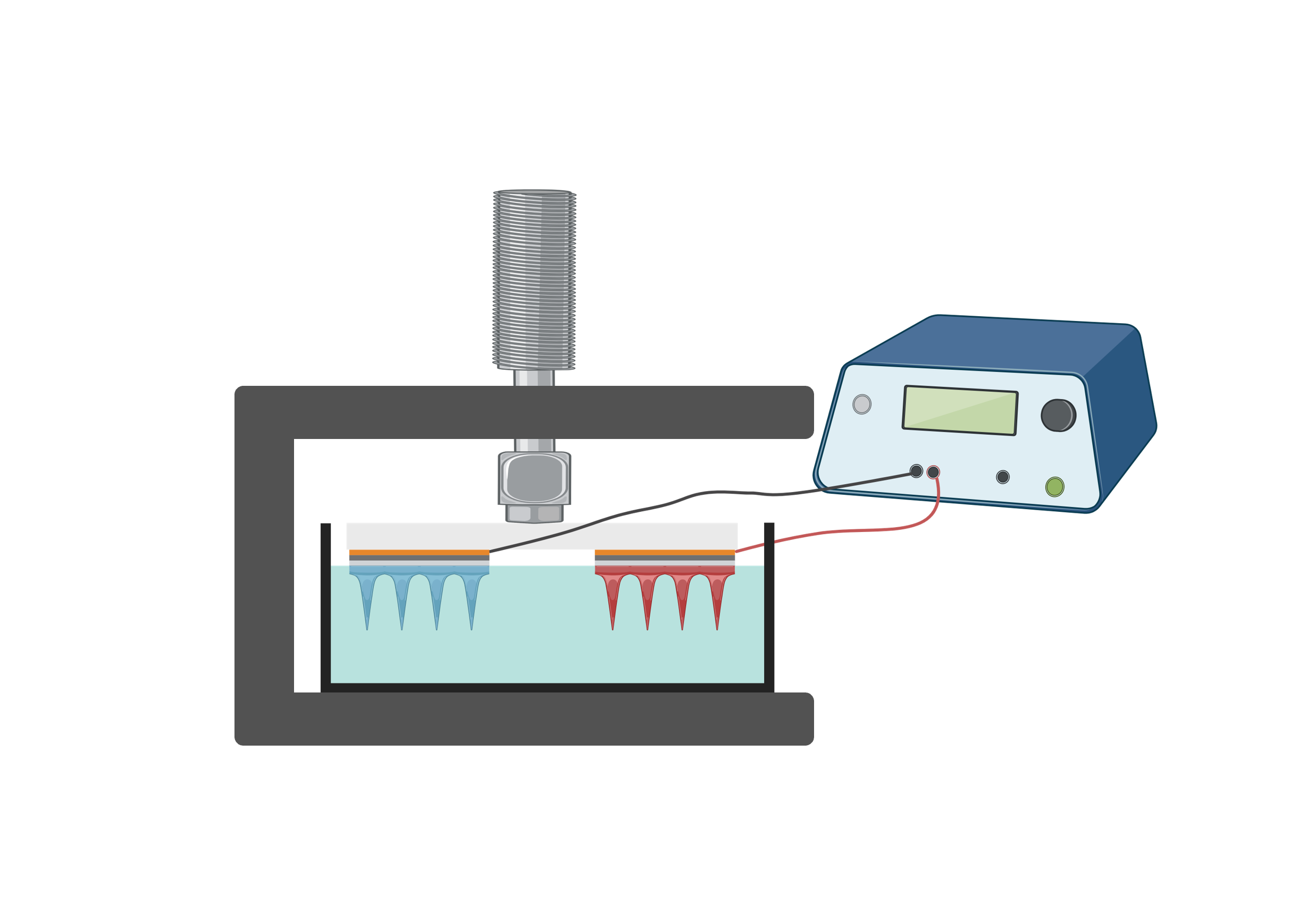

Supplement: Supplementary 1 — Figs. S1 to S15 Table S1 [file bmef.0044.f1.zip › FS2 .png]

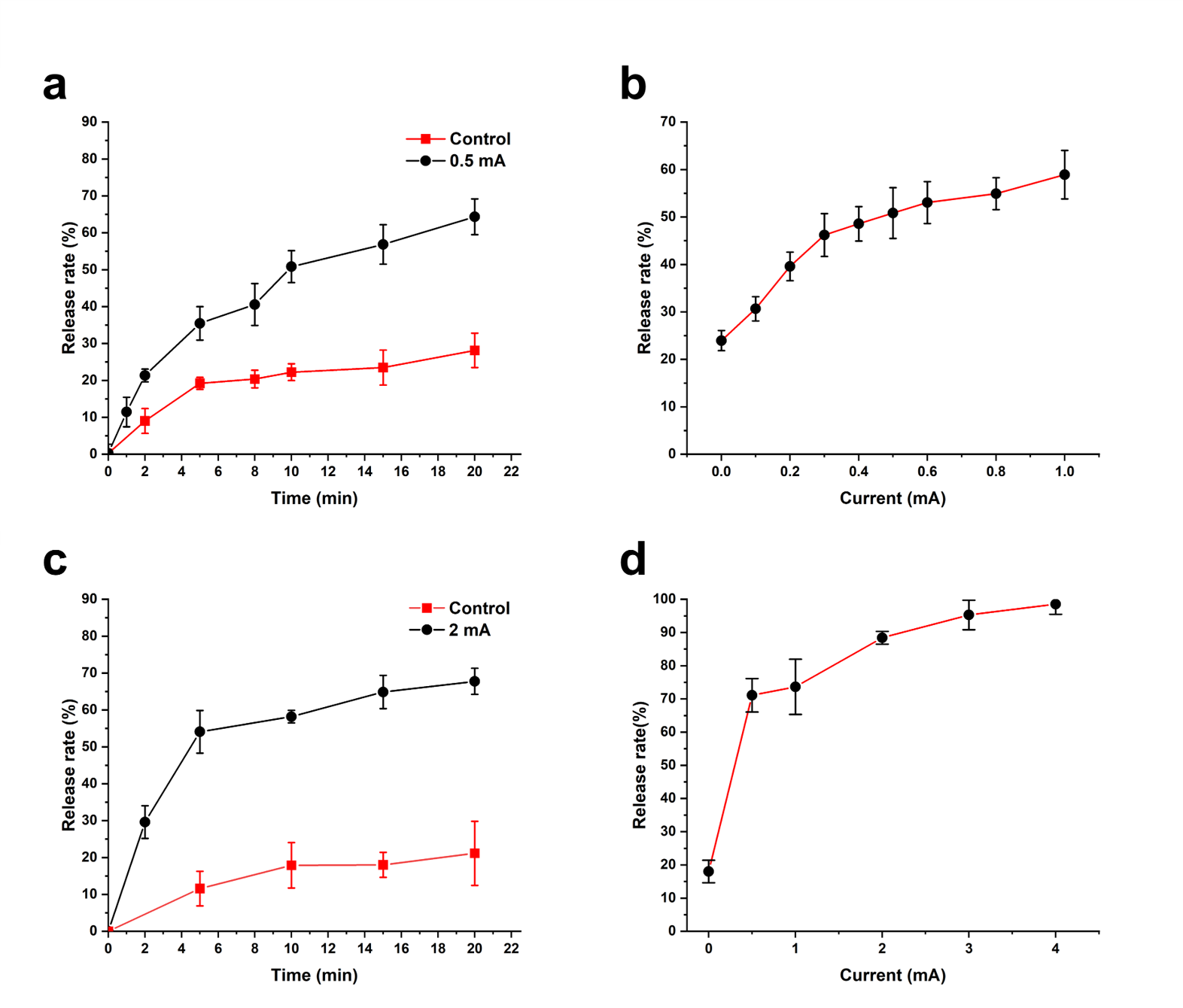

Supplement: Supplementary 1 — Figs. S1 to S15 Table S1 [file bmef.0044.f1.zip › FS3 .png]

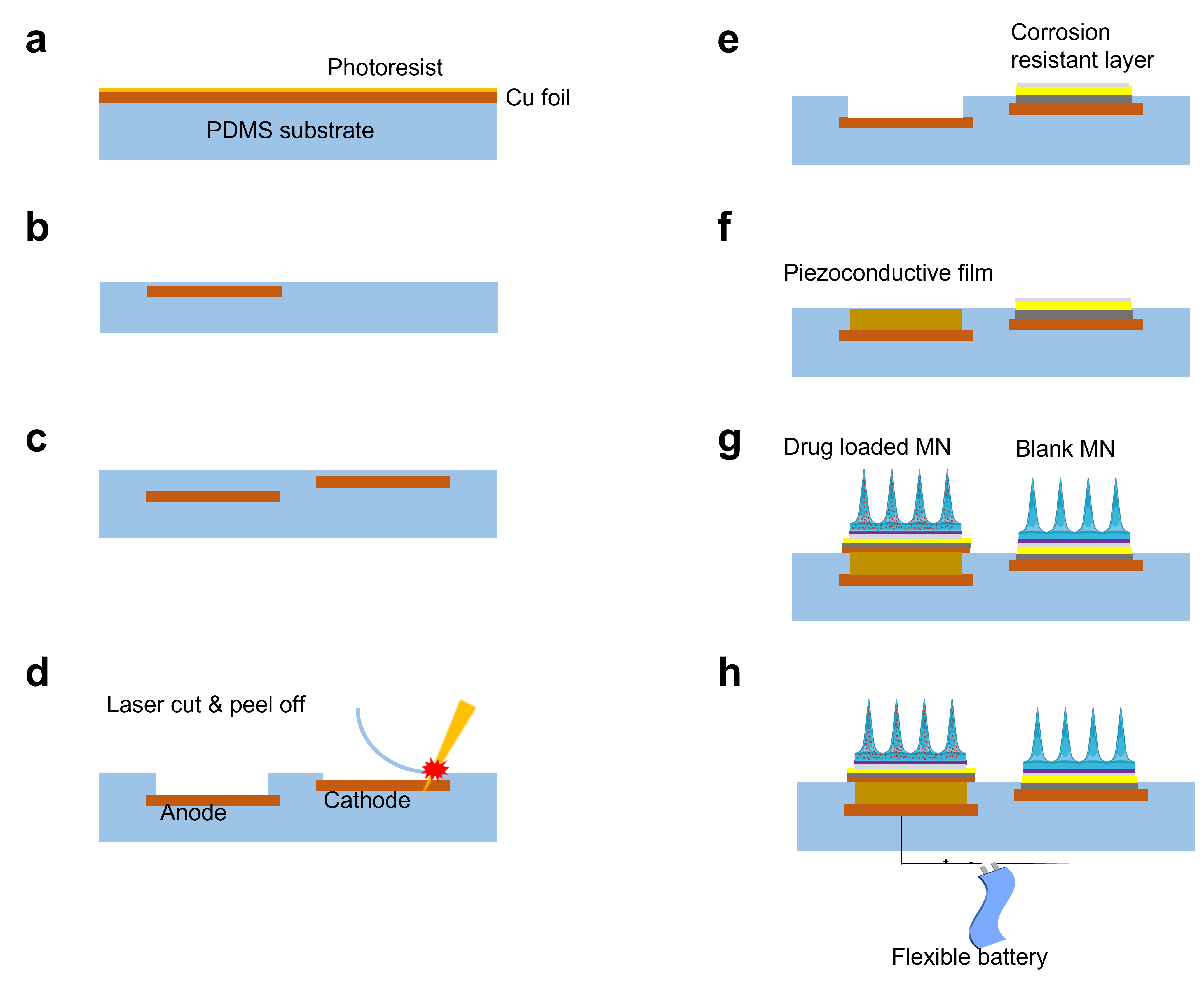

Supplement: Supplementary 1 — Figs. S1 to S15 Table S1 [file bmef.0044.f1.zip › FS4 .png]

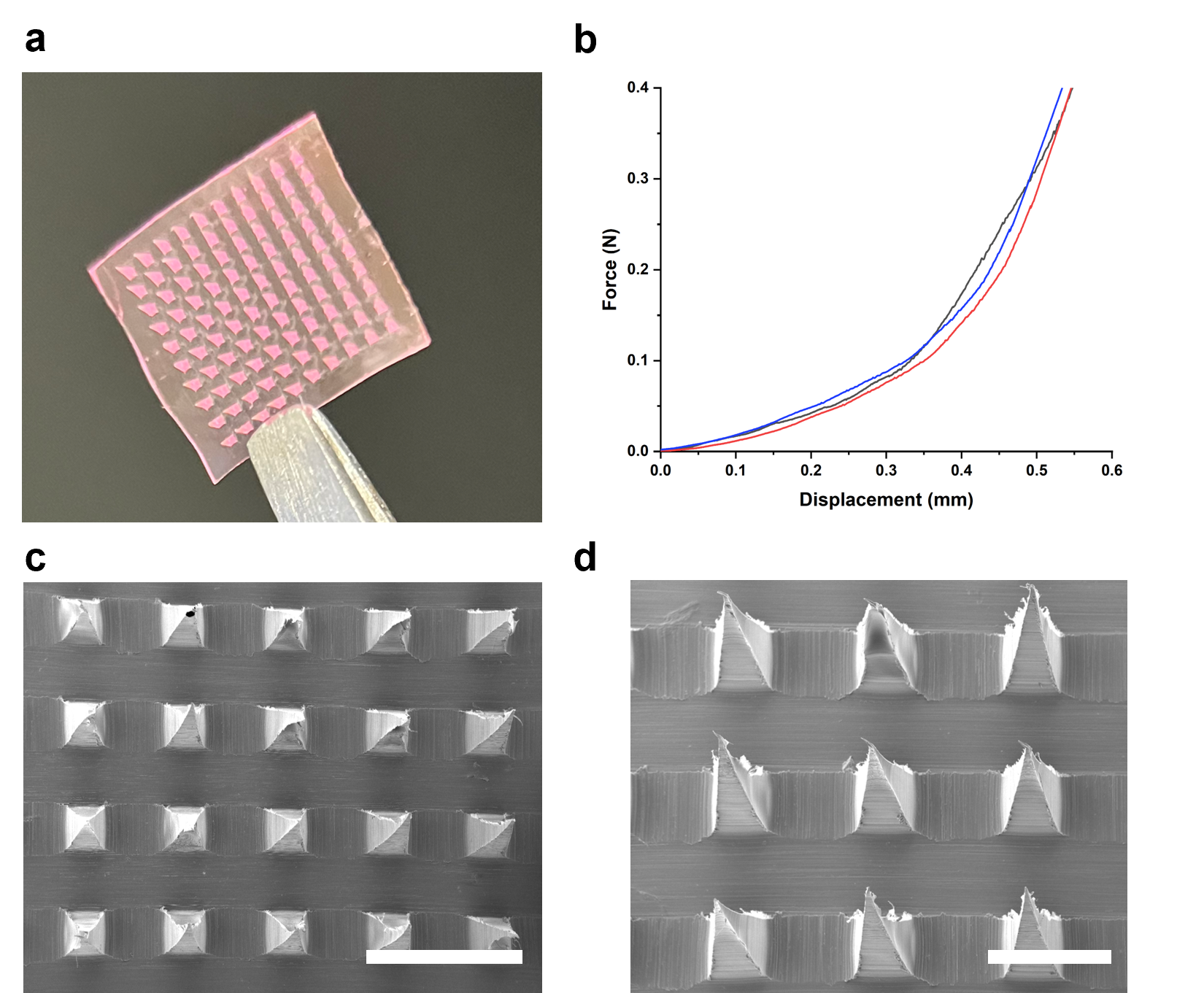

Supplement: Supplementary 1 — Figs. S1 to S15 Table S1 [file bmef.0044.f1.zip › FS5 .png]

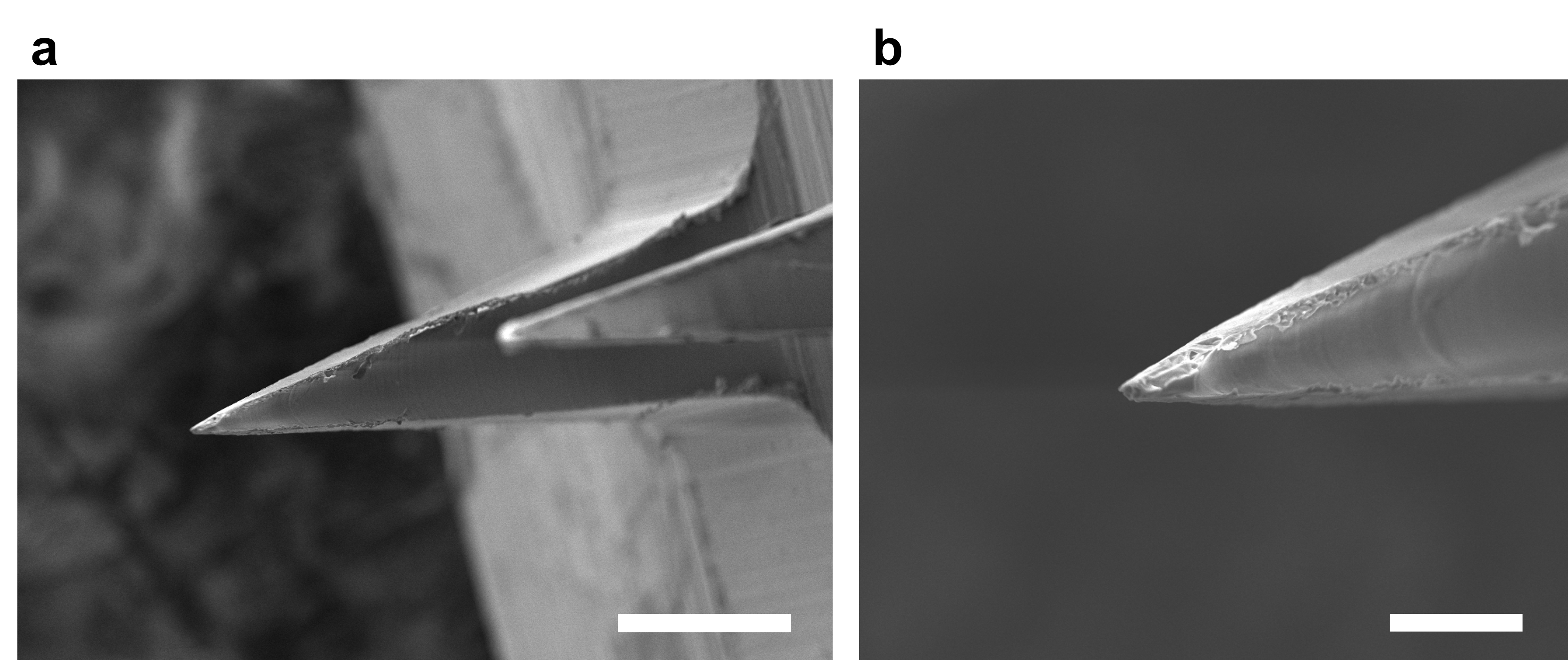

Supplement: Supplementary 1 — Figs. S1 to S15 Table S1 [file bmef.0044.f1.zip › FS6.png]

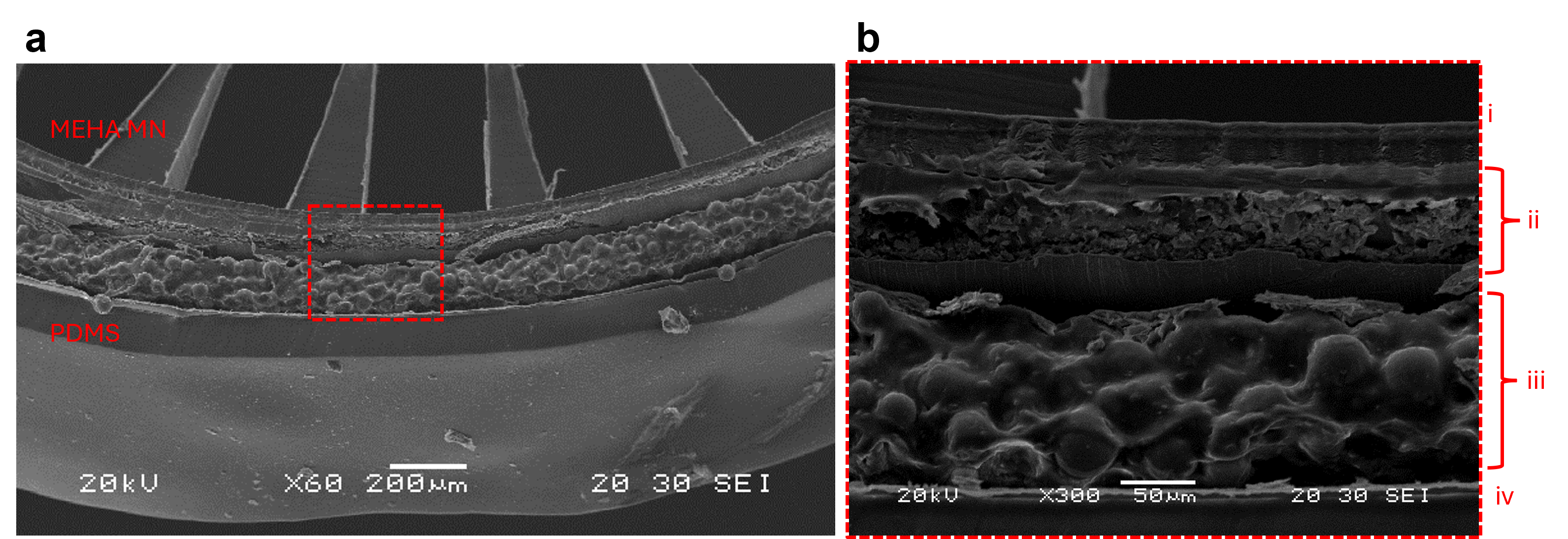

Supplement: Supplementary 1 — Figs. S1 to S15 Table S1 [file bmef.0044.f1.zip › FS7 .png]

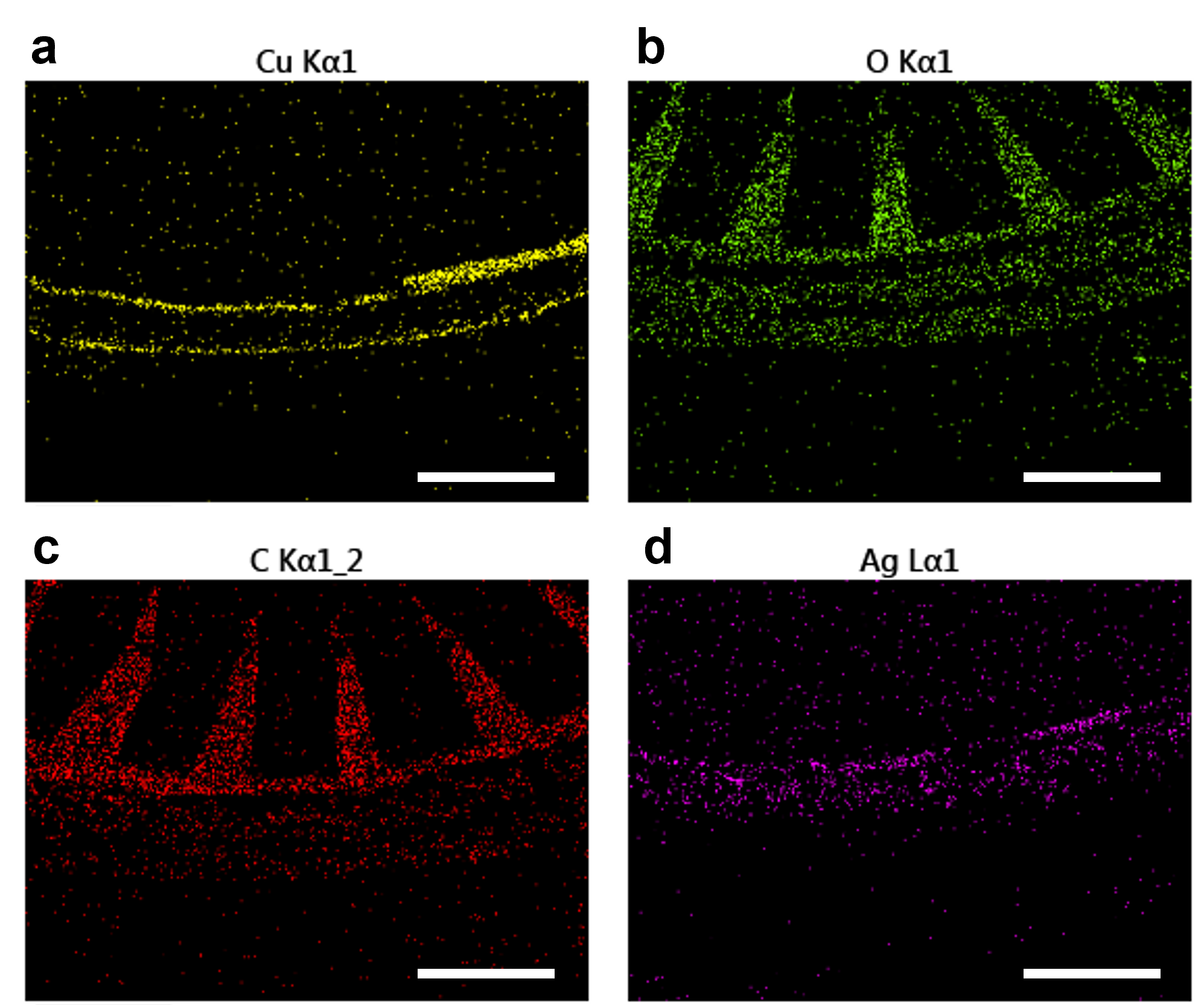

Supplement: Supplementary 1 — Figs. S1 to S15 Table S1 [file bmef.0044.f1.zip › FS8 .png]

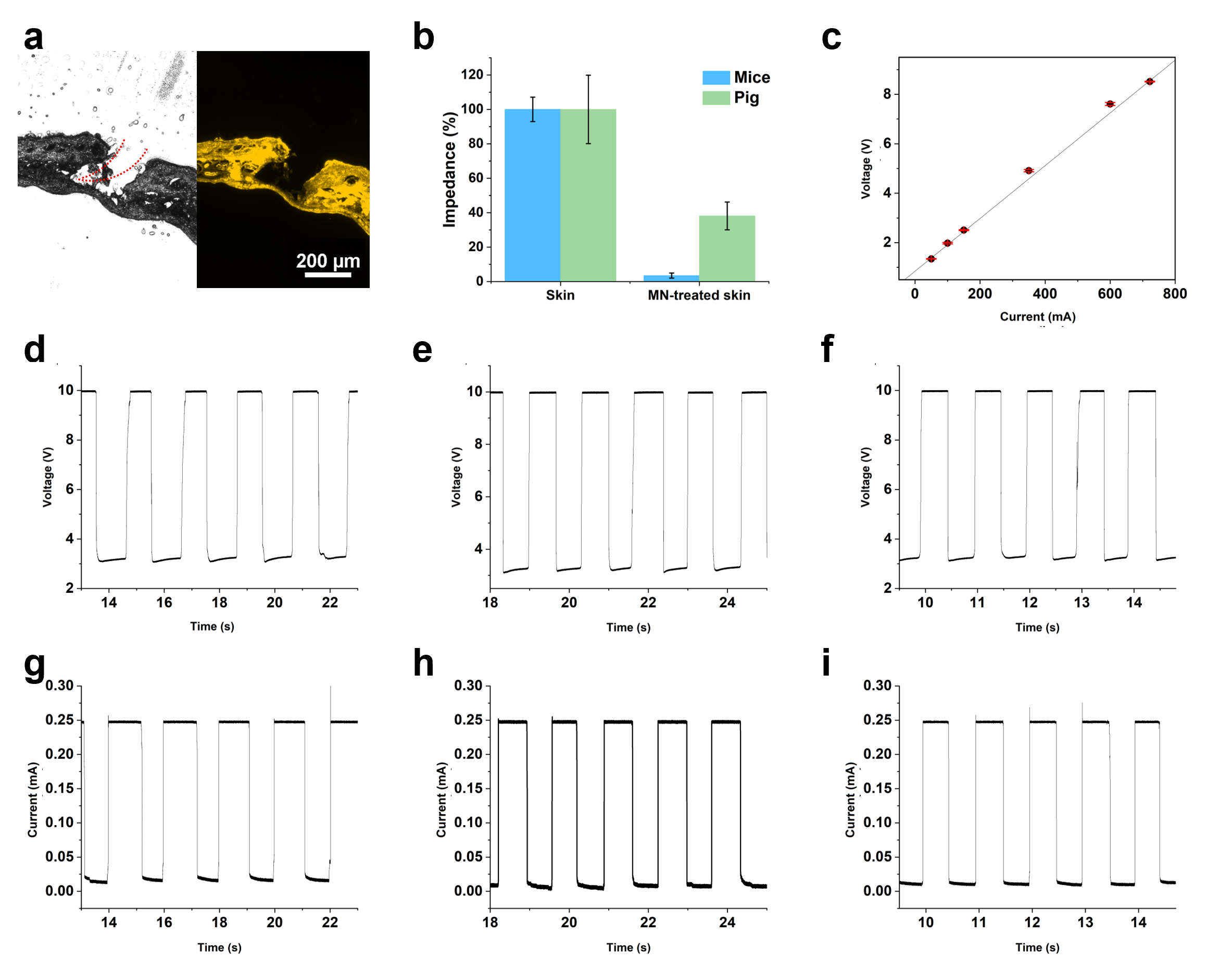

Supplement: Supplementary 1 — Figs. S1 to S15 Table S1 [file bmef.0044.f1.zip › FS9 .png]
